# Enhanced Immune Response Against the Thomsen-Friedenreich Tumor Antigen Using a Bivalent Entirely Carbohydrate Conjugate

**DOI:** 10.3390/molecules25061319

**Published:** 2020-03-13

**Authors:** Kristopher A. Kleski, Kevin R. Trabbic, Mengchao Shi, Jean-Paul Bourgault, Peter R. Andreana

**Affiliations:** 2801 West Bancroft Street, Department of Chemistry and Biochemistry and School of Green Chemistry and Engineering, The University of Toledo, Toledo, OH 43606, USA; kkleski@rockets.utoledo.edu (K.A.K.); ktrabbi@gmail.com (K.R.T.); broad.mengchaoshi@gmail.com (M.S.); jpb6337@gmail.com (J.-P.B.)

**Keywords:** cancer vaccine, tumor associated carbohydrate antigen, zwitterionic polysaccharide, C-type lectin receptor, multivalent vaccine

## Abstract

The Thomsen-Friedenreich (TF) antigen is a key target for the development of anticancer vaccines, and this ongoing challenge remains relevant due to the poor immunogenicity of the TF antigen. To overcome this challenge, we adopted a bivalent conjugate design which introduced both the TF antigen and the Thomsen-nouveau (Tn) antigen onto the immunologically relevant polysaccharide A1 (PS A1). The immunological results in C57BL/6 mice revealed that the bivalent, Tn-TF-PS A1 conjugate increased the immune response towards the TF antigen as compared to the monovalent TF-PS A1. This phenomenon was first observed with enzyme-linked immunosorbent assay (ELISA) where the bivalent conjugate generated high titers of IgG antibodies where the monovalent conjugate generated an exclusive IgM response. Fluorescence-activated cell sorting (FACS) analysis also revealed increased binding events to the tumor cell lines MCF-7 and OVCAR-5, which are consistent with the enhanced tumor cell lysis observed in a complement dependent cytotoxicity (CDC) assay. The cytokine profile generated by the bivalent construct revealed increased pro-inflammatory cytokines IL-17 and IFN-γ. This increase in cytokine concentration was matched with an increase in cytokine producing cells as observed by ELISpot. We hypothesized the mechanisms for this phenomenon to involve the macrophage galactose *N*-acetylgalactosamine specific lectin 2 (MGL2). This hypothesis was supported by using biotinylated probes and recombinant MGL2 to measure carbohydrate-protein interactions.

## 1. Introduction

The Thomsen-Friedenreich (TF, β-d-Gal*p*-(1,3)-α-d-GalNAc*p*) antigen is a tumor associated carbohydrate antigen (TACA) that has significant roles in the progression of carcinomas of the breast, colon, prostate, liver, and more [1]. High expression levels of the TF antigen on tumor cells is positively correlated to poor prognosis, and an increased ability to metastasize [2,3]. The relationship between TF expression and metastasis has been definitively shown to be mediated through galectin-3 on endothelial cells, where selective pressure to non-TF expressing tumor populations can decrease metastasis [4,5]. Furthermore, patients that were able to develop endogenous anti-TF antibodies have significantly improved prognosis [6,7]. These characteristics of the TF antigen coupled to the fact that the TF antigen is not exposed on normal, healthy tissue makes the TF antigen a viable target for immunotherapy [8,9]. Regardless, strategies to develop potent immune responses to the TF antigen have been thwarted by its seemingly low immunogenicity [10,11,12,13,14,15,16].

Our research efforts focus on the issues of developing carbohydrate specific immunity by utilizing a semi-synthetic entirely carbohydrate vaccine platform. This two-component platform consists of an anomeric aminooxy TACA derivative conjugated to Polysaccharide A1 (PS A1) via oxime bond and has been demonstrated with the TF antigen, the Thomsen-nouveau (Tn, α-d-GalNAc*p*) antigen, and the sialyl-Tn (STn, α-d-Neu5Ac-(2,6)-α-d-GalNAc*p*) antigen [17,18,19]. PS A1 is a zwitterionic polysaccharide (ZPS) isolated from the commensal bacteria *Bacteroides fragilis* (ATCC 25285/NCTC 9343) capable of activating CD4+ T cells in a major histocompatibility complex-II (MHC-II) dependent mechanism [20,21]. PS A1 is an effective immunogenic carrier for TACAs and is evidenced by our previous work with Tn-PS A1 and STn-PS A1 [17,18,22]. These PS A1 conjugates were able to induce protective immune responses that consisted of potent antibody production, cellular immunity, and the production of proinflammatory markers such as T helper 17 (Th17) cells and IL-17, which are essential in the protection against cancer [23,24].

Another elegant strategy to improve carbohydrate immunogenicity involves targeting innate immune receptors on the surface of antigen presenting cells (APCs) [25,26,27]. C-type lectin receptors (CLRs), such as the macrophage galactose *N*-acetylgalactosamine specific lectin 2 (MGL2, CD301b) have been investigated for their potential to influence immune responses [28,29,30,31,32]. The major ligand for MGL2 is the Tn glycan, and reports suggest the Tn glycan has direct positive influence on antigen uptake mediated in an MGL2 dependent mechanism by dendritic cells (DCs) that lead to CD4+ T cell activation [33,34,35,36,37]. Studies by Leclerc et al. have validated this phenomenon, demonstrating a correlation between an increased Tn density on MUC6 and other peptides to enhance antigen internalization by APCs [38]. Antigen internalization was markedly increased when compared to the non-glycosylated peptides, which was believed to be a consequence of MGL2 interaction with Tn. These responses were also associated with an increase in Th2 related cytokines, Th17 related cytokines, and an increased expansion of B cell populations within germinal centers [38,39]. Van Kooyk et al. have also reported increased MGL2 dependent antigen internalization and presentation of Tn containing antigens. These responses lead to Th1 polarization and increased IFN-γ production coupled with enhanced cross-presentation ultimately activating antigen specific CD4+ T cells and CD8+ T cell responses [40,41].

Based on our current understanding of carbohydrate immunity, we sought to create an immunogen with the ability to increase an immune response to the TF antigen without the need to generate unnatural epitopes on the TF structure which requires additional synthetic steps and antibody cross-reactivity to the natural TF epitope. The inclusion of the Tn antigen into the TF-PS A1 conjugate may induce an “adjuvant-like” affect by recruiting key components in the development of an immune response, such as MGL2, and the subsequent activation of the adaptive immune response involving both T and B cells. Herein, we report the synthesis of the unimolecular, bivalent Tn-TF-PS A1 conjugate and its immune efficacy as compared to the TF-PS A1 and Tn-PS A1 conjugates.

## 2. Results and Discussion

### 2.1. Synthesis and Characterization of TACA-PS A1 Conjugates

**Scheme 1:**
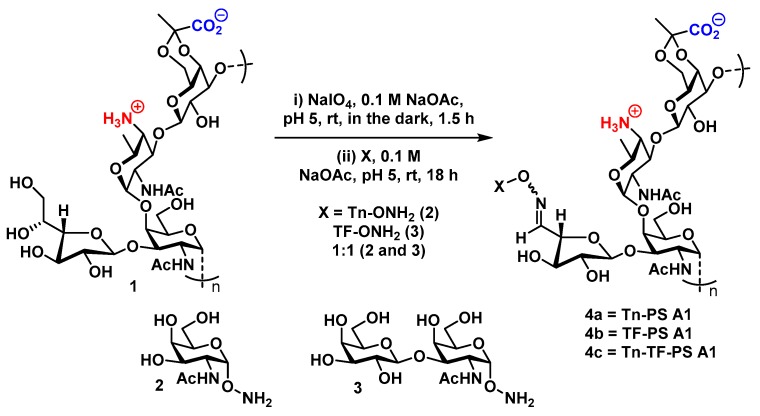
Synthesis of polysaccharide A1 (PS A1) conjugates.

The synthesis of TACA-PS A1 conjugates Tn-PS A1 (**4a**) [18], TF-PS A1 (**4b**) [19], and Tn-TF-PS A1 (**4c**) were achieved when sodium periodate was used to regioselectively oxidize the terminal vicinal diol on the d-galactofuranose moiety on the repeating unit of PS A1 using a 0.1 M acetate buffering system at a pH of 5. Periodate oxidations form a cyclic, five-membered ring intermediate in which terminal vicinal diols will undergo oxidative cleavage faster than cyclic trans-diols [42]. To avoid oxidative cleavage of the furanose ring, 0.5 equivalents of sodium periodate was used per repeating unit of PS A1 to ensure regioselectivity. The resulting aldehyde was then exposed to the anomeric aminooxy derivatives of Tn (**2**), TF (**3**), and a 1:1 molar ratio of **2** and **3** to form the oxime conjugates in Tn-PS A1 (**4a**), TF-PS A1 (**4b**), and Tn-TF-PS A1 (**4c**), respectively as shown in Scheme 1. Purification of these conjugates was achieved through dialysis with a 10 kDa molecular weight cut off followed by lyophilization of the dialyzed material. The resulting white solid was analyzed by proton and COSY NMR spectroscopy to confirm chemical transformations which are evidenced by: (1) oxime proton signal (7.99–8.00 ppm), (2) new anomeric proton signals (~5.77 ppm), and (3) additional *N*-acetyl peaks (2.29–2.31 ppm) (Doc. S1). An estimated percent loading can be obtained from the proton spectra of Tn-PS A1 (**4a**), TF-PS A1 (**4b**), and Tn-TF-PS A1 (**4c**) by integration of the *N*-acetyl peaks. Although this method may introduce potential error due to peak overlap, we have demonstrated this method previously [17,43]. Based on the integration of *N*-acetyl peaks, percent loadings were calculated to be 13% for Tn-PS A1 (**4a**), 19% for TF-PS A1 (**4b**), and a combined loading of 18% for Tn-TF-PS A1 (**4c**) (Table S1).

### 2.2. ELISA Reveals Enhanced Antibody Production Against the TF antigen with the Tn-TF-PS A1 Conjugate

Conjugates Tn-PS A1, TF-PS A1, and Tn-TF-PS A1 were evaluated for their immunological potency in conjunction with either the depot adjuvant TiterMax^®^ Gold (TMG) [44,45] or the monophosphoryl lipid A (MPLA) containing adjuvant Sigma Adjuvant System^®^ (SAS) [44,46]. Groups of five Jax C57BL/6 mice were immunized intraperitoneally four times in biweekly intervals when using TMG or were immunized intraperitoneally three times in triweekly intervals when SAS was used. To determine antibody production against the native antigens, antibody titers were determined by enzyme-linked immunosorbent assay (ELISA) with 96-well plates coated with either glycoconjugate Tn-bovine serum albumin (Tn-BSA) or TF-BSA (Scheme S1).

**Figure 1:**
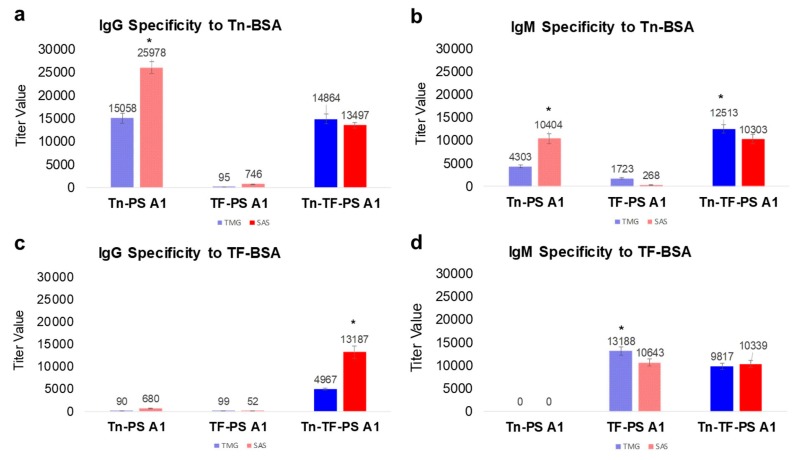
Serum polyclonal antibody specificity to Thomsen-nouveau-bovine serum albumin (Tn-BSA) or Thomsen-Friedenreich (TF)-BSA conjugates. Groups of mice (n = 5) were immunized with tumor associated carbohydrate antigen (TACA)-PS A1 conjugates (**4a**, **4b**, or **4c**) in conjunction with TiterMax^®^ Gold (TMG) (blue bars) or Sigma Adjuvant System^®^ (SAS) (red bars). ELISA experiments were performed in triplicate. (**a**) IgG specificity towards Tn-BSA. (**b**) IgM specificity towards Tn-BSA. (**c**) IgG specificity towards TF-BSA. (**d**) IgM specificity towards TF-BSA. Both PS A1 and PBS served as controls in our murine immunization studies and there was no cross-reactivity observed to either Tn-BSA or TF-BSA. Error bars are derived from variations in response and are shown as mean ± standard error of mean. Significance is noted between adjuvant groups TMG and SAS. Where * *p* < 0.05 was obtained by two tailed Student’s *t*-test.

Figure 1a displays IgG titers as an average from individual mice against Tn-BSA. Antiserum from monovalent Tn-PS A1 immunized mice exhibited high IgG titers when SAS (red bar) and TMG (blue bar) was used as an adjuvant. The antibody response to Tn-PS A1 had increased IgG isotype populations as compared to the IgM isotype (Figure 1b). This response is an indication of T cell activation, immunological memory, and affinity maturation which are desirable outcomes in cancer immunotherapies. Figure 1c depicts IgG titers against TF-BSA and most notably, monovalent TF-PS A1 immunized mice did not showcase significant IgG binding events. The humoral response against TF-PS A1 remained exclusively within the IgM isotype (Figure 1d) and was independent of adjuvant used. The contrast in these ELISA data between conjugates Tn-PS A1 and TF-PS A1 correspond to the low immunogenicity of the native TF antigen. Individual mouse titer values obtained from immunizations in conjunction with SAS are illustrated in Figure S1.

As shown in Figure 1a–d mice immunized with Tn-TF-PS A1 developed high IgG and IgM titers for both Tn-BSA and TF-BSA. The most significant result shown in Figure 1c provides evidence of an enhanced antibody production and maturation against the TF antigen. Mice immunized against TF-PS A1 did not generate significant IgG titers to the TF antigen, but high IgG titers were developed when mice were immunized with the bivalent conjugate Tn-TF-PS A1. We also detected minimal antibody cross reactivity from mice immunized with the monovalent conjugates Tn-PS A1 and TF-PS A1 which suggested that is not the likely source for the development of the IgG titers as observed in Figure 1c. These ELISA data indicated that the addition of Tn onto the TF-PS A1 construct augmented the immune response towards the TF antigen. To rule out immune activation from differential particle sizes by individual aggregation characteristics, PS A1 (1) and conjugates Tn-PS A1, TF-PS A1, and Tn-TF-PS A1 were examined with dynamic light scattering (DLS) [47]. These experiments showed that conjugates Tn-PS A1, TF-PS A1, and Tn-TF-PS A1 have similar hydrodynamic radii and polydispersity indexes (Figure S2).

### 2.3. Polyclonal Antibodies Bind Human Tumor Cell Lines

**Figure 2:**
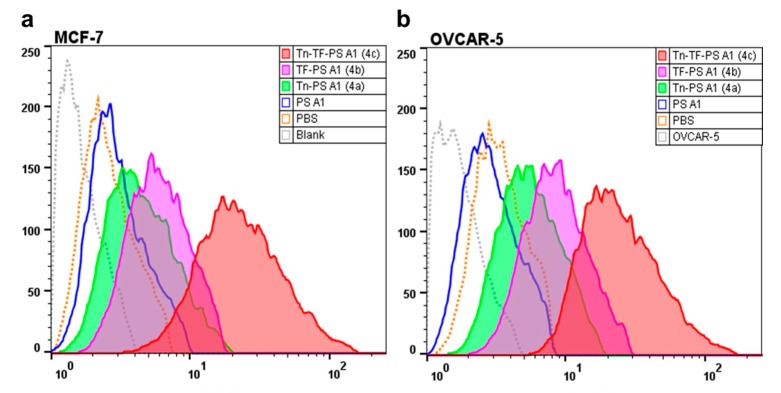
Flow cytometry study with antiserum obtained from mice immunized with **1**, **4a**, **4b**, or **4c** and human tumor cell lines. (**a**) MCF-7 human breast tumor cell line. (**b**) OVCAR-5 human ovarian tumor cell line.

Antiserum was further evaluated with flow cytometry to determine polyclonal antibody binding to human tumor cells MCF-7 [48] (Figure 2a), OVCAR-5 [49,50] (Figure 2b), and normal human breast tissue MCF-10A [51] (Figure S3). The anti-serum of Tn-TF-PS A1 demonstrated a 97% gated-shift in fluorescently sorted cell populations compared to MCF-7 cells alone. This result is expected as the MCF-7 cell line is known to express both the Tn and the TF antigens [48]. However, similar binding events were seen using human ovarian tumor cell line OVCAR-5 with antiserum from Tn-TF-PS A1 giving a 98% shift in fluorescently sorted cell populations. We also note in Figure S3 that there is negligible binding of antiserum from TACA-PS A1 constructs to MCF-10A.

### 2.4. Polyclonal Antibodies Mediate Tumor Cell Killing with Complement

**Figure 3:**
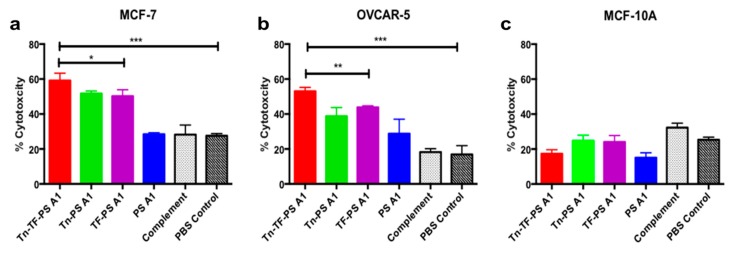
Antibody mediated complement dependent cytotoxicity (CDC) with antiserum obtained from mice immunized with **1**, **4a**, **4b**, or **4c** in combination with rabbit complement. (**a**) MCF-7 cell line. (**b**) OVCAR-5 cell line. (**c**) MCF-10A cell line. Experiments were performed in triplicate. Error bars are shown as mean ± standard error of mean. Where * *p* < 0.05, ** *p* < 0.005, *** *p* < 0.0005 were obtained by two tailed Student’s *t*-test.

Antibody function was then assessed using a complement dependent cytotoxicity (CDC) assay where antiserum derived from native PS A1, Tn-PS A1, TF-PS A1, or Tn-TF-PS A1 were compared. This experiment also utilized antiserum derived from PBS to evaluate nonspecific antibody interactions with the target cells, as well as a complement control to evaluate nonspecific killing of tumor cells. In Figure 3a, antiserum from Tn-TF-PS A1 exhibited 59% cytotoxicity towards the MCF-7 cell line which was statistically significant in comparison with antiserum from TF-PS A1. Additionally, antiserum from Tn-TF-PS A1 had 53% cytotoxicity towards OVCAR-5 (Figure 3b) and was also statistically significant when compared to antiserum from TF-PS A1. Interestingly, there is no significant difference between the observed cytotoxicity from antisera derived from monovalent constructs Tn-PS A1 and TF-PS A1, which seems to be in contrast to the data in Figure 1. We hypothesized these small variations in cytotoxicity to be a result of the similar IgM responses of Tn-PS A1 and TF-PS A1, as the C1q protein in complement binds IgM Fc portions 1000-fold more than the IgG Fc portions [52]. In all cases, we observed negligible cytotoxicity to the non-carcinoma MCF-10A cell line as a control (Figure 3c). Collectively, we observed that antiserum from Tn-TF-PS A1 had significant cytotoxicity over the antiserum derived from the monovalent counterpart TF-PS A1.

### 2.5. Quantification of Cytokines Released from Splenocytes In Vitro

**Figure 4:**
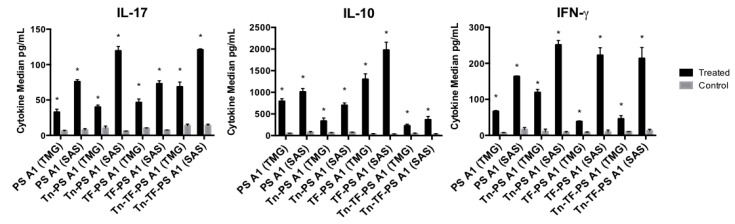
IL-17, IL-10, and IFN-γ in vitro cytokine production assay from stimulated murine splenocytes. PS A1 and TACA-PS A1 constructs were used as stimuli. The cytokines were detected using sandwich ELISA and experiments were performed in triplicate. Error bars are shown as mean ± standard error of mean. Where * *p* < 0.05 was obtained by using two-tailed Student’s *t*-test.

Figure 4 illustrates an investigation between Th17, Treg, and Th1 related cytokines activated by native PS A1, Tn-PS A1, TF-PS A1, or Tn-TF-PS A1. PS A1 and its conjugates have been documented to influence the activation of Th17 cells, which are known to produce IL-17 [22] and can assist in antibody production [53]. Notably, co-culture of construct Tn-TF-PS A1 and the splenocytes harvested from mice immunized with Tn-TF-PS A1, revealed an increase in IL-17 by 59% when compared to PS A1. Similarly, immunization with Tn-PS A1 resulted in an increase in IL-17 by 57%. However, IL-17 production resulting from immunization with TF-PS A1 was decreased. The pattern of IL-10 quantification was opposite to that of IL-17, peaking with the construct TF-PS A1 and minimized with Tn-TF-PS A1. IL-10 is a major regulatory cytokine associated with Tregs [54]. The observed high level of IL-10 matched with a low level of IL-17 for TF-PS A1 is a possible explanation for the poor IgG development seen when immunizations were done with TF-PS A1 in conjunction with either TMG or SAS. Finally, we observed IFN-γ production in splenocytes from mice immunized with Tn-PS A1, TF-PS A1, and Tn-TF-PS A1 were increased when compared to **1** alone. The increase in IFN-γ may be attributed to MPLA, which is known to stimulate Th1 polarization [55].

### 2.6. Quantification of Cytokine Producing Cells with ELISpot

**Figure 5:**
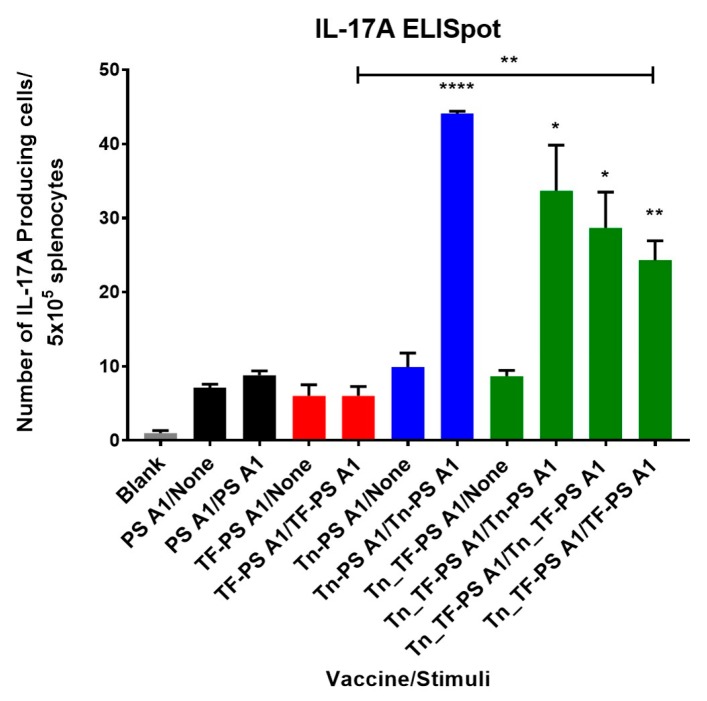
IL-17 ELISpot response from unstimulated and stimulated murine splenocytes obtained from mice immunized against the respective TACA-PS A1 constructs. Error bars are shown as mean ± standard error of mean response between 3 mice, in which, each mouse was performed in triplicate. Where * *p* < 0.05, ** *p* < 0.01, **** *p* < 0.0001 by using two-tailed Student’s *t*-test between stimulated and unstimulated splenocytes.

In conjunction with cytokine quantification via sandwich ELISA, we performed a cellular based ELISpot assay to quantify cytokine producing cells. The cytokine IL-17 is solely produced by Th17 cells, and this relationship was exploited to show differences in CD4+ T cell activation between constructs PS A1, Tn-PS A1, TF-PS A1, and Tn-TF-PS A1 [22,23,24]. Figure 5 summarizes the number of Th17 producing cells developed from a splenocyte culture derived from mice immunized against the respective stimuli. The largest development of IL-17 producing cells was observed with Tn-PS A1, which showed a five-fold increase in IL-17 producing cells when compared to splenocytes derived from native PS A1. Conversely, TF-PS A1 developed even less IL-17 producing cells than PS A1 although it was not a significant change. To determine the influence conjugate Tn-TF-PS A1 had on IL-17 producing cells, anti-Tn-TF-PS A1 splenocytes were co-cultured with either Tn-PS A1, TF-PS A1, or Tn-TF-PS A1. The highest count of IL-17 producing cells in this group was observed when Tn-PS A1 was used as the stimulus. Alternatively, when TF-PS A1 conjugate TF-PS A1 was used to stimulate anti-Tn-TF-PS A1 splenocytes a three-fold increase of IL-17 producing cells was observed as compared to anti-TF-PS A1 splenocytes stimulated with the same construct. These data support the self-adjuvanting effect Tn can produce when incorporated as a bivalent construct, increasing immunity towards the TF antigen. Specificity was also supported by data presented in Figure S4 where the stimulation of anti-Tn-PS A1 splenocytes with TF-PS A1 did not increase IL-17 production.

IFN-γ is a canonical cytokine produced by Th1 cells, and the amount of IFN-γ producing cells in an ELISpot assay can provide insight into T cell activation [17,56,57]. Displayed in Figure S5, anti-Tn-TF-PS A1 splenocytes were able to generate increased levels of IFN-γ producing cells than anti-TF-PS A1 splenocytes when both were co-cultured with TF-PS A1. This result further supports an increased cellular response against the TF antigen with the bivalent Tn-TF-PS A1 conjugate. In addition to quantifying IL-17 and IFN-γ producing cells, we also quantified IL-10 producing cells (Figure S6). Interestingly, the increase in IL-10 producing cells when anti-Tn-TF-PS A1 splenocytes were stimulated with TF-PS A1 was not as a significant increase when compared to the previous cytokine producing cells.

### 2.7. Biotinylated Conjugate Probes Bind to Recombinant MGL2

**Figure 6:**
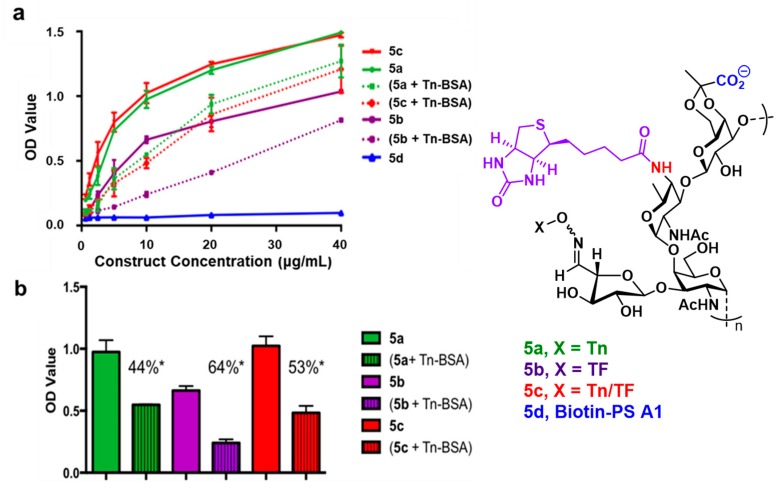
(**a**) MGL2 binding assay using probes **5a**, **5b**, **5c**, and **5d** (Scheme S2). (**b**) Percent inhibition by Tn-BSA in presence of **5a**, **5b**, **5c**, and **5d**. Experiments were performed in triplicate. Error bars are shown as mean ± standard error of mean. * denotes % inhibition by Tn-BSA.

To identify MGL2 as a potential mediator for the observed increase in immune activation by construct Tn-TF-PS A1, four biotinylated conjugate probes (**5a**, **5b**, **5c**, and **5d**, Scheme S2) were synthesized. These probes were constructed by reacting the primary amine of the 2,4-dideoxy-4-amino-d-*N*-acetyl-fucose (AAT) constituent saccharide on the PS A1 repeating unit with sulfo-NHS-biotin. The probes (**5a**, **5b**, **5c**, and **5d**) were used in an assay where recombinant MGL2 was coated on 96-micro-well plates and streptavidin-alkaline phosphatase was used to detect binding interactions (Figure 6). The results indicated that probes **5a** and **5c** showed sufficient binding to MGL2 (Figure 6a). **5b** also gave a positive response but to a lesser extent than probes **5a** and **5c**. Constructs **5a**, **5b**, **5c**, and **5d** (10 μg mL^−1^) were also observed to be competitively inhibited by Tn-BSA (10 μg mL^−1^) where **5b** experienced the greatest inhibition (Figure 6b). MGL2 binding was negligible for the negative control **5d**.

## 3. Materials and Methods

### 3.1. Vaccinations with TiterMax^®^ Gold or Sigma Adjuvant System^®^

Jax C57BL/6 male mice (6 weeks) were obtained from Jackson Laboratories and maintained by the Department of Laboratory Animal Resources (DLAR) at the University of Toledo. All animal protocols were approved and performed in compliance with the relevant laws and institutional guidelines set forth by the Institutional Animal Care and Use Committee (IACUC) of the University of Toledo (protocol number 107956). Individual Tn-, TF-, and Tn-TF-PS A1 constructs were mixed in a 1:1 ratio of 50 μL of TiterMax^®^ Gold or 1:1 ratio of 100 μL SAS to achieve a final concentration of 20 μg for TACA-PS A1 constructs and injected into 7-week-old C57BL/6 mice. Groups of mice (n = 5) were immunized by intraperitoneal injections (IP) on day 0, 14, 28, 42 for TMG or on day 0, 21, 42 for SAS. Blood sera were obtained using a cardiac puncture technique on day 52.

### 3.2. Enzyme Linked Immunosorbent Assay (ELISA)

Either Tn- or TF-BSA was coated on Immulon^®^ Microtiter™ 4 HBX 96 well-plates using 3 μg mL^−1^ in 0.1 M carbonate buffer (pH 9.2) and incubated for 18 h at 4 °C. Plates were washed three times with 200 μL of washing buffer (1× PBS buffer with 0.05% Tween^®^ 20 (*v*/*v*)) and blocked with 200 μL of 3% BSA (*w*/*v*) for 1 h at room temperature. Serum from mice were initially diluted at 1:100 and then serially half-log_10_ diluted for a final volume of 100 μL in each well, and then incubated for 2 h at 37 °C. After incubation, the plates were washed three times with 200 μL of washing buffer. Alkaline phosphatase-linked secondary antibodies (anti-IgM and anti-IgG) were used to detect primary antibodies bound to either Tn- or TF-BSA. The procedure for the secondary anti-IgM (Southern Biotech) antibodies called for a 1:1000 dilution and anti-IgG antibodies (Jackson ImmunoResearch) were diluted (1:5000). A total of 100 μL of secondary antibody was placed in wells and incubated for 1 h at 37 °C. The plates were washed three times with 200 μL of washing buffer and *p*-nitrophenyl phosphate (PNPP) (1 mg mL^−1^) in diethanolamine buffer (pH 9.8) was added at 100 μL per well and incubated for 30 min. The optical density was read at 405 nm using a BioTek PowerWave HT Microplate Spectrophotometer. All assays were performed in triplicate. Titers were determined by regression analysis with dilutions plotted against absorbance. The titer cutoff value was set at 0.2 which was two times the control PBS anti-serum for titer determination. Statistical analysis from ELISAs for experimental groups were compared with the controls using paired *t* test and GraphPad Prism 6.

### 3.3. Flow Cytometry

MCF-7, OVCAR-5, and MCF-10A were cultured in 10% FBS RPMI 1640. 1.0 × 10^6^ cells of each cell line were incubated at 4 °C for 1 h in the dark with 1:50 dilution of the following separate antiserums: 1× PBS control, **1**, **4a**–**4c**. The cells were washed three times in 250 μL of FACS buffer (2% FBS (*v*/*v*) in 1× PBS, 0.001% sodium azide (*w*/*v*)) by centrifuging at 1000 rpm. 100 μL Anti-IgG Alexa Fluor^®^ 488 (1:50 dilution) was added to the cells and incubated at 4 °C in the dark for 1 h followed by three washes with 250 μL of FACS staining buffer. The cells were fixed with freshly prepared 1% paraformaldehyde (*v*/*v*) and analyzed using BD Biosciences FACS Calibur at the University of Toledo Core Flow Cytometry Facility. FlowJo analysis software was used to process flow cytometry data.

### 3.4. Complement Dependent Cytotoxicity Assay

MCF-7 cells (1.0 × 10^4^) and OVCAR-5 cells (1.0 × 10^4^) were seeded in 96 well plates and incubated overnight in a 5% CO_2_ incubator at 37 °C. The plates were then washed with 2% BSA (*w*/*v*) in DPBS. After washing, 100 μL of experimental antiserum solutions, diluted 1:20 in DPBS, were added to the corresponding wells and incubated for 1 h. The experimental wells were washed and incubated with 10% rabbit complement (Pel-Freez) for 1 h at 37 °C. The control values of the LDH assay kit (Cytotoxicity Detection Kit; Roche, Mannheim, Germany) were determined from spontaneous LDH release (low control) and 1% Triton X-100 (*v*/*v*) (high control) and incubated for 1 h at 37 °C. 50 μL of cell supernatant was transferred to a new 96 well-plate containing 50 μL of DPBS. According to manufacturer protocol, 100 μL of the colorimetric LDH detection reagent was added to each well and the O.D. was read at 490 nm. The percentage cellular cytotoxicity was calculated using the following equation: Cell cytotoxicity % = (experimental values – low control values)/(high control values – low control values) × 100.

### 3.5. MGL2 Binding Assay

Mouse recombinant MGL2 (R&D systems) 2.5 μg mL^−1^ was used to coat Immulon^®^ Microtiter™ 4 HBX 96 well-plates in 1× PBS buffer (with CaCl_2_/MgCl_2_) pH 7.2 for 18 h at 4 °C. The plates were then washed with 200 μL of 1× PBS washing buffer (with CaCl_2_/MgCl_2_ and 0.05% Tween 20 (*v*/*v*)) three times. Biotin-PS A1 and respective biotinylated conjugates **4a**–**4c** were serially diluted from 40–0.625 μg mL^−1^ and incubated for 2 h at 37 °C in 1× DPBS with CaCl_2_/MgCl_2_. Plates were then washed with 200 μL of 1× PBS washing buffer three times. A streptavidin-alkaline phosphatase (Sigma Aldrich) solution was diluted (1:1000) and 100 μL was added to each well and incubated for 1 h at 37 °C. The plates were washed three times with 200 μL of 1× PBS washing buffer and then PNPP (1 mg mL^−1^) in diethanolamine buffer (pH 9.8) was added at a 100 μL per well and incubated for 30 min. The optical density was determined at 405 nm. Percent inhibition by Tn-BSA followed the same procedure, however, 10 μg mL^−1^ of Tn-BSA was co-incubated with **4a**–**4c** before binding competition to MGL2 was attempted. Percent inhibition was calculated using the equation: [(O.D. of **4a**–**4c** binding to MGL2) − (O.D. of co-incubation of 4a–c with Tn-BSA)/(O.D. of **4a**–**4c** binding to MGL2)] × 100.

### 3.6. Other Methods

Additional methodology is included in Doc. S5-Materials and Methods.

## 4. Conclusions

Tn-PS A1 was observed to be consistent in mounting an IgG immune response towards the Tn antigen whether TMG or SAS was used. However, using TF-PS A1 to elicit immunity towards the TF antigen provided unsatisfactory results. Figure 1 indicated that the use of either TMG or SAS in conjunction with TF-PS A1 had little effect on IgG development, which is a critical intimation of T cell help and B cell maturation. When PS A1 was conjugated with both Tn and TF, there was a profound difference in proliferation of anti-TF IgGs when compared to the monovalent TF-PS A1 construct. Notably, polyclonal antibodies obtained from Tn-PS A1, TF-PS A1, and Tn-TF-PS A1 immunizations were assessed to bind Tn-BSA or TF-BSA. These BSA conjugates permitted the focus of antibody specificity towards either Tn or TF and not PS A1 as the carrier.

To further suggest an adjuvant-like effect with the Tn glycan, we observed an increase in pro-inflammatory cytokines and pro-inflammatory cytokine producing cells noted in Figure 4 and Figure 5 respectively. The increased pro-inflammatory environment is also suggested by the enhanced IgG response from Tn-TF-PS A1, enhanced tumor cell recognition in FACS (Figure 2), and increased tumor cell killing as noted in the CDC assay (Figure 3).

To determine if MGL2 was a potential target, four biotinylated probes (**5a**, **5b**, **5c**, and **5d**) were evaluated in a colorimetric assay. It was determined that Tn-biotin-PS A1 (**5a**) and Tn-TF-biotin-PS A1 (**5c**) had similar binding profiles, which in part, support the claim that the inclusion of the Tn antigen promotes a greater interaction with MGL2. MGL2 has been documented to have a lower affinity towards TF, which was confirmed in the observations noted in Figure 6 [29,31]. These experiments, in conjunction with competitive binding, suggested the affinity of the TF epitope is less for MGL2. These data support our hypothesis and the inclusion of the Tn epitope in Tn-TF-PS A1 permitted greater interactions with MGL2 which led to enhanced antigen uptake and antigen presentation by APCs. However, further experiments are required to definitively determine the mechanisms of immune activation.

The development of a semi-synthetic, bivalent Tn-TF-PS A1 construct has led to an increase in immunogenicity for the TF antigen as observed with ELISA, flow cytometry, CDC, and cytokine production. Literature precedent suggests that the increase in immune response is attributed to the MGL2 receptor which led to a more efficient uptake of the Tn-TF-PS A1 construct [33,38,39,40]. This stands in contrast to other unimolecular multivalent constructs for which there was no enhanced response towards an individual TACA. [58]. However, we did observe a similarity to unimolecular multivalent constructs where the seemingly immunodominant epitope was suppressed. This simple model seems applicable to multiple vaccine platforms which may include peptides, proteins, nanoparticles, and lipids leading to an increase in the therapeutic potential of carbohydrate-based vaccines.

## Supplementary Materials

The following are available online, Doc.S1–S5.
