# Supplementary material for: Enhanced Immune Response Against the Thomsen-Friedenreich Tumor Antigen Using a Bivalent Entirely Carbohydrate Conjugate
Source: Molecules. 2020 Mar 13;25(6):1319. doi: 10.3390/molecules25061319 (PMC7144725; doi:10.3390/molecules25061319)
Supplement: Supplementary file 1 [file molecules-25-01319-s001.zip › SI/Doc. S1-Compound Characterization.docx]

**Characterization**

^1^H NMR/^13^C NMR of (**8**).

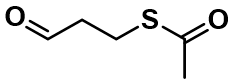

^1^H NMR/^13^C NMR of (**9**).

**
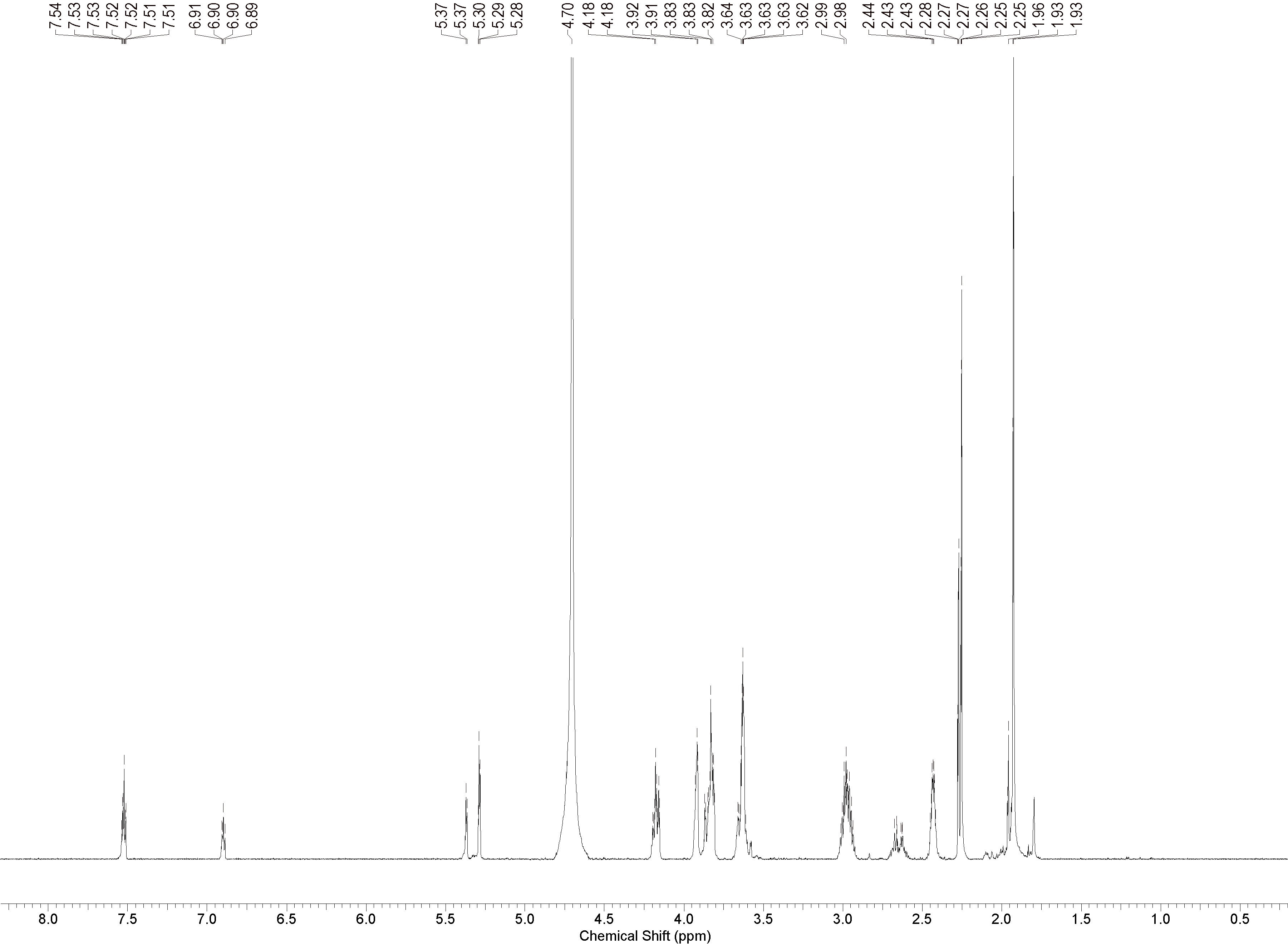
**

***
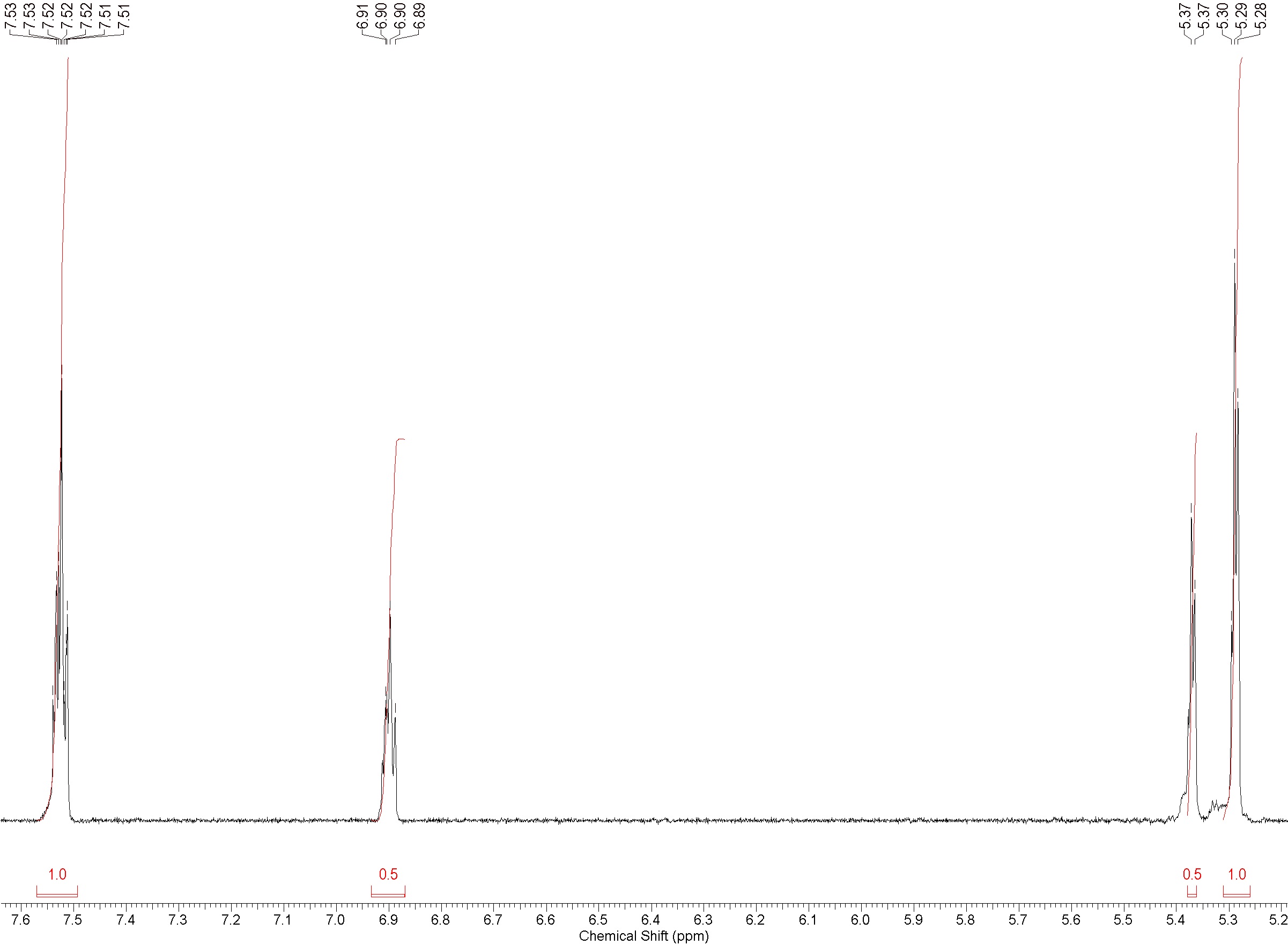
***

*E*

*Z*

***
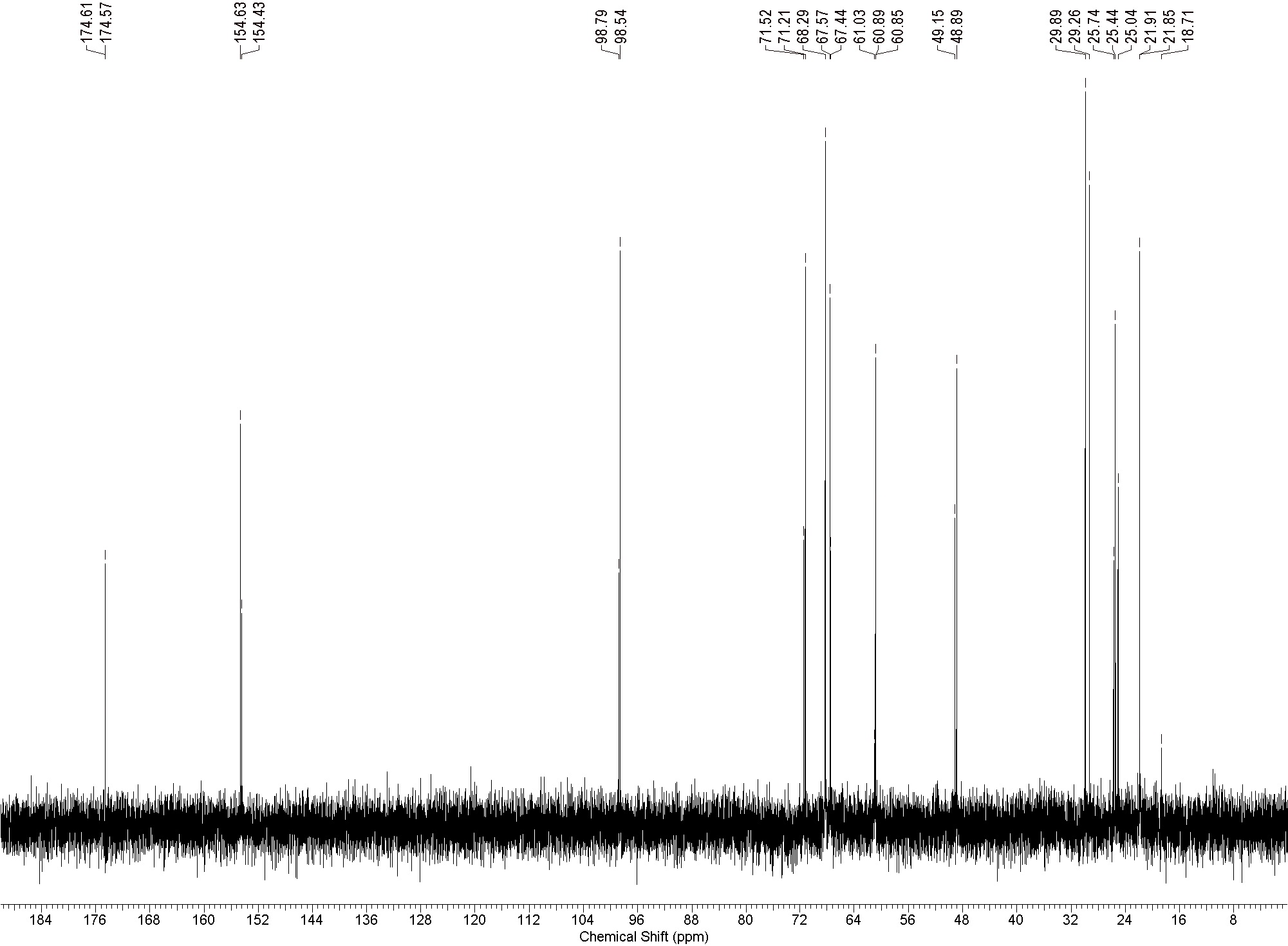
***

^1^H NMR/^13^C NMR of (**10**).

**
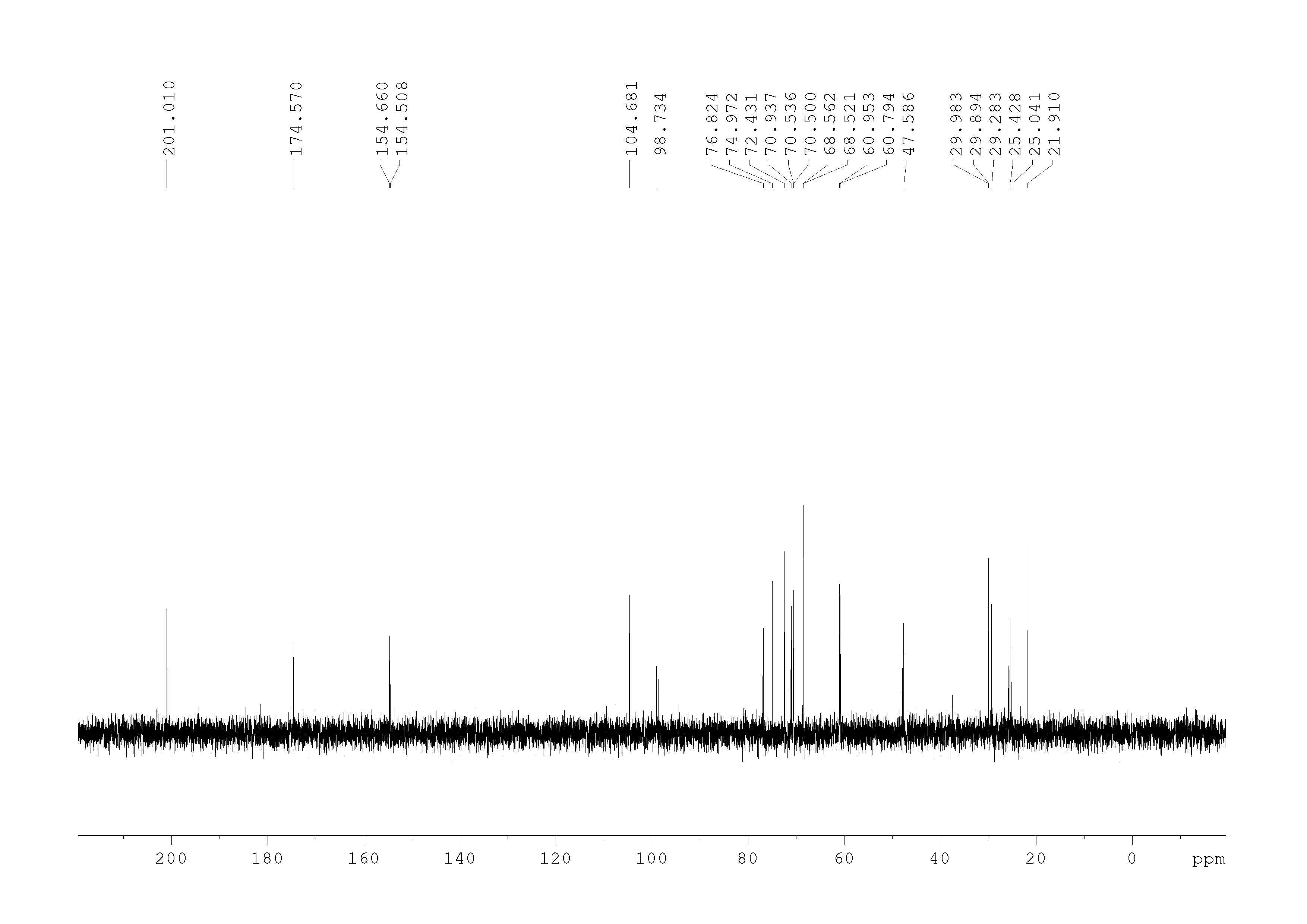
**

MALDI-TOF OF BSA Maleimide (**11**).


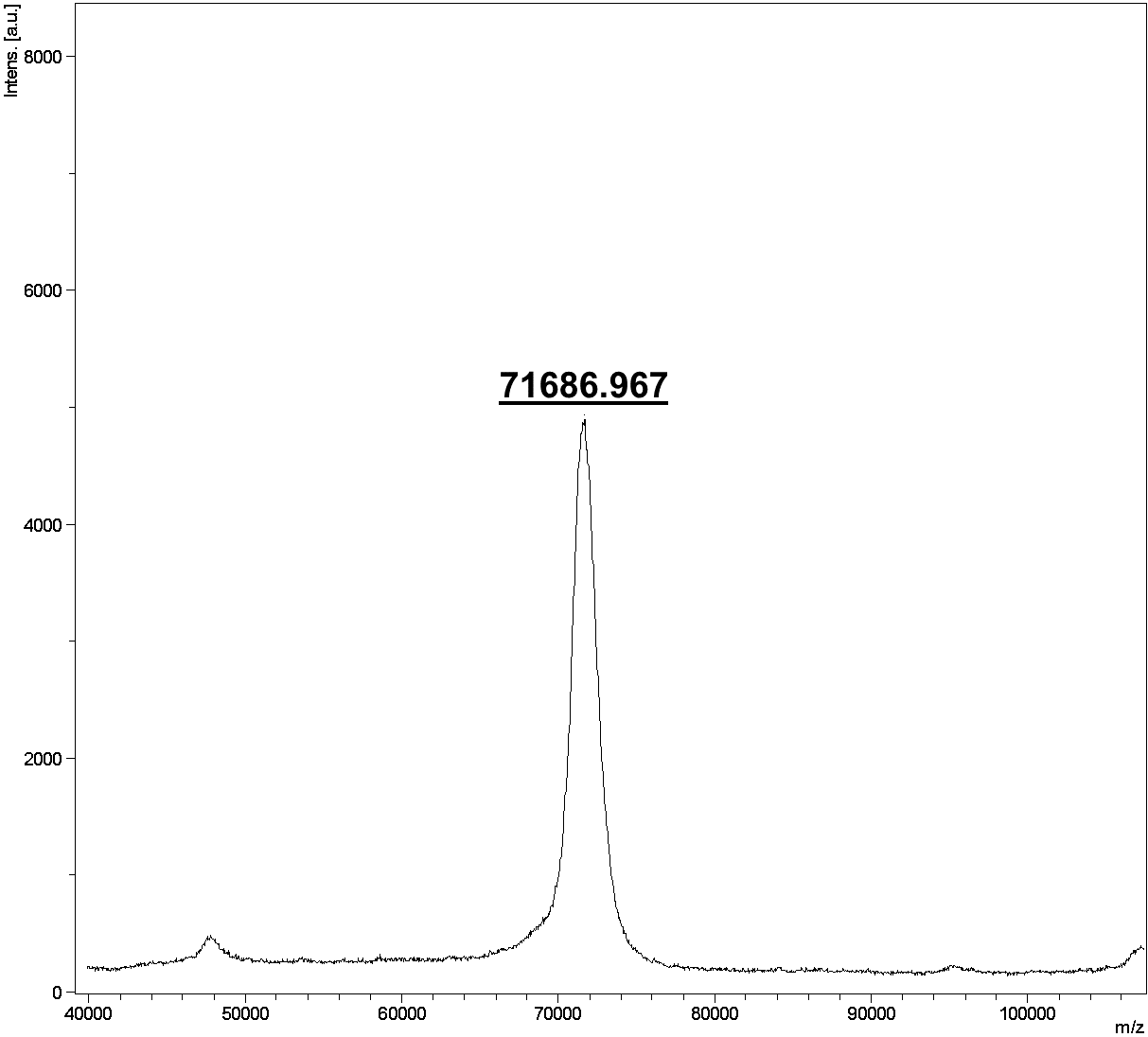


MALDI-TOF OF Tn-BSA (**12**).


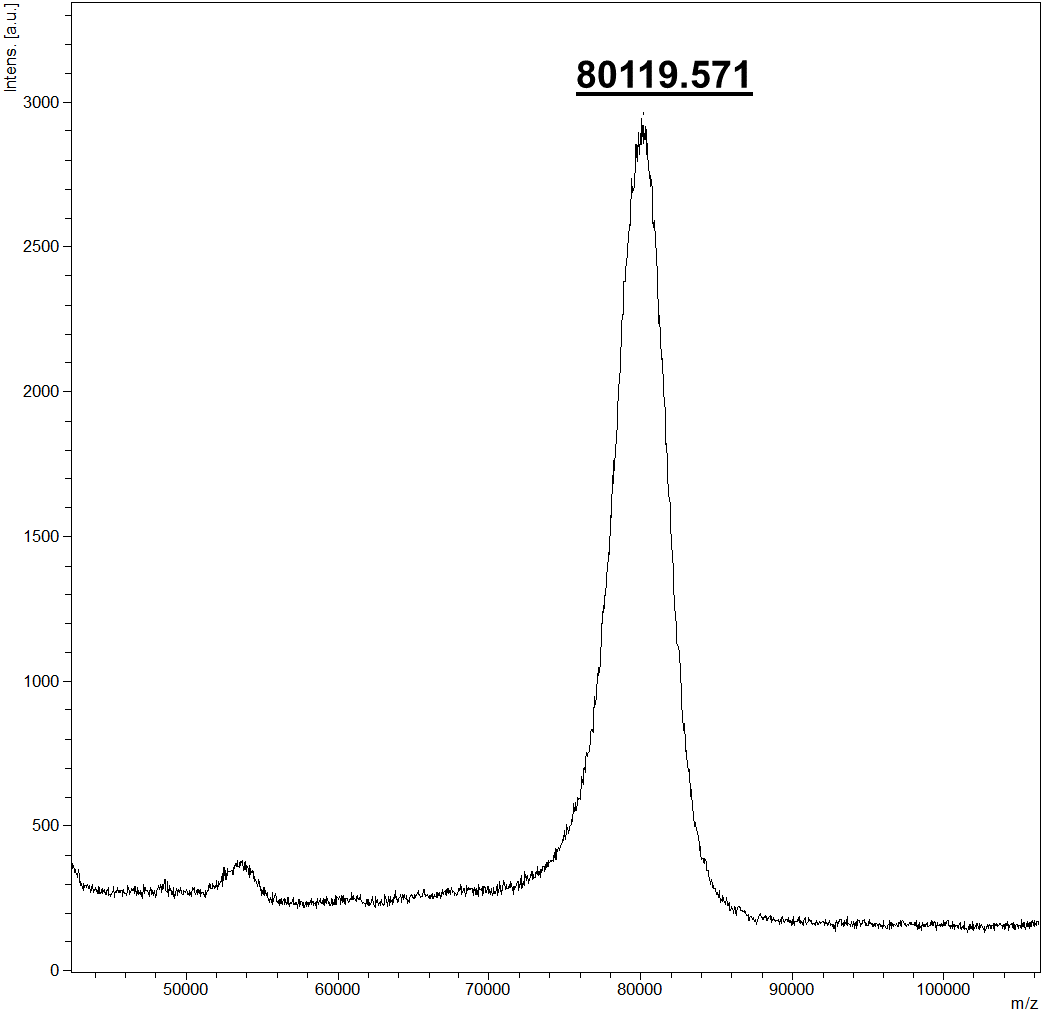


MALDI-TOF OF TF-BSA (**13**).


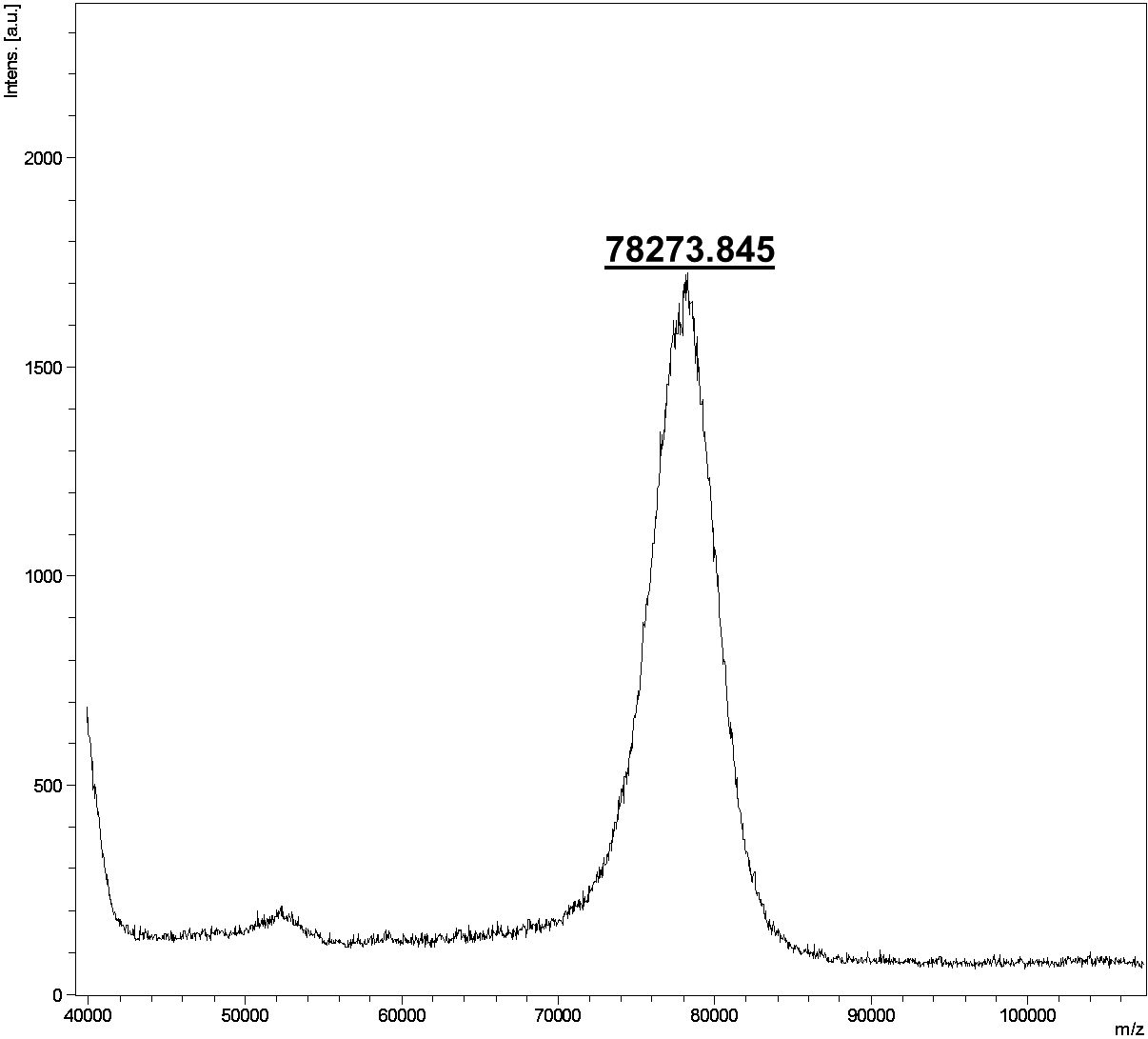


***
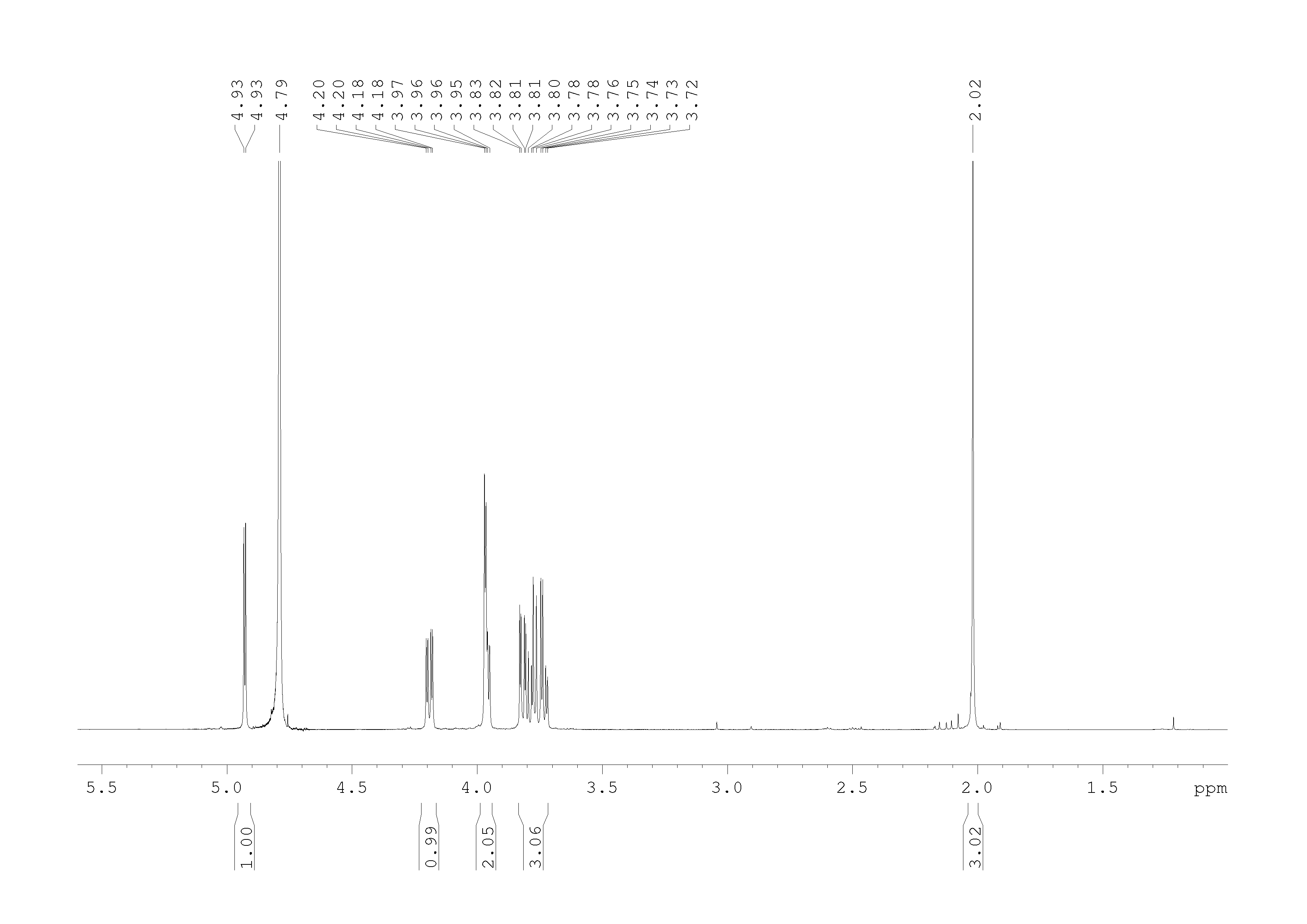
***^1^H NMR Spectra of Tn-ONH_2_ (**2**).

***
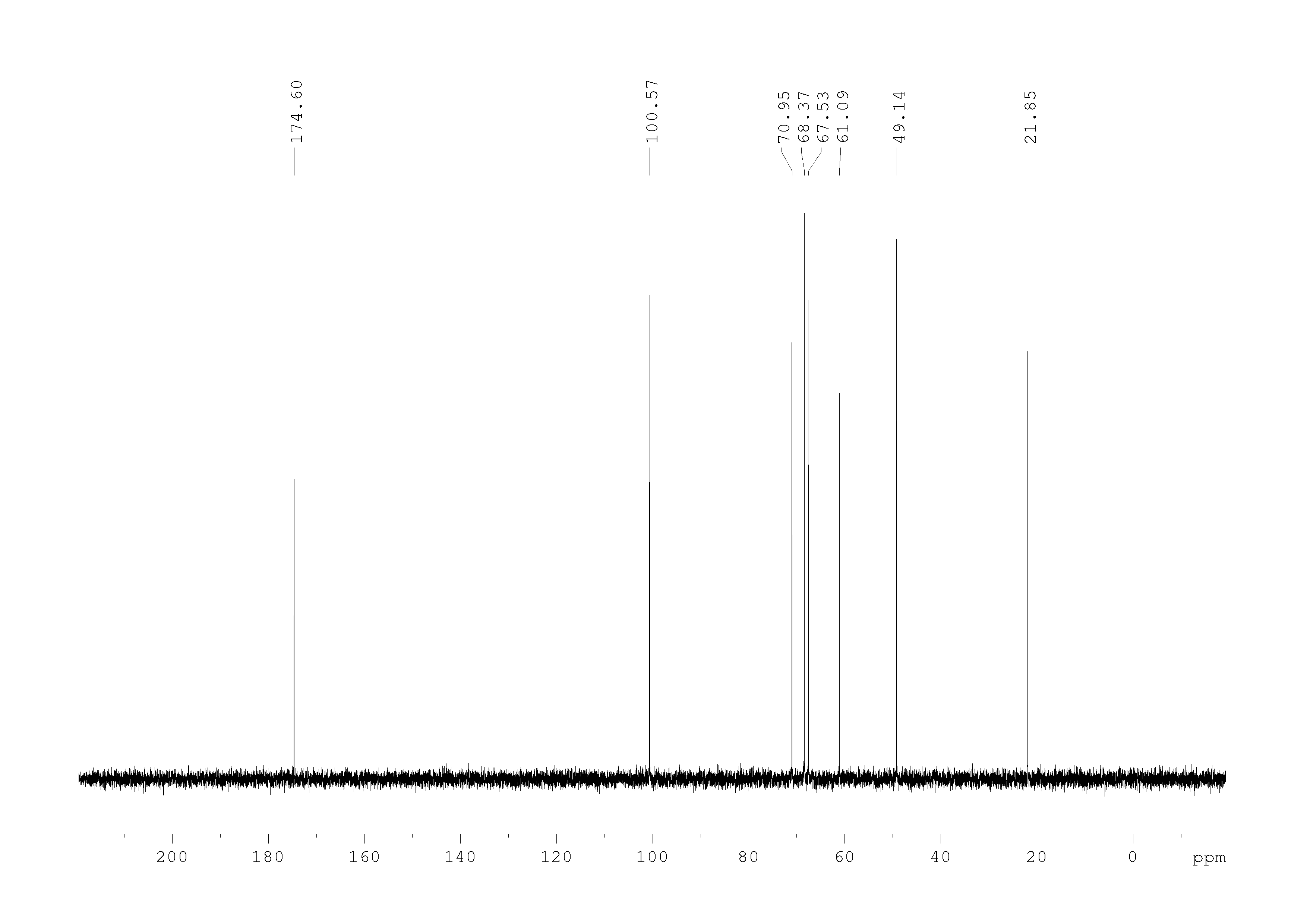
***^13^C NMR Spectra of Tn-ONH_2_ (**2**).

***
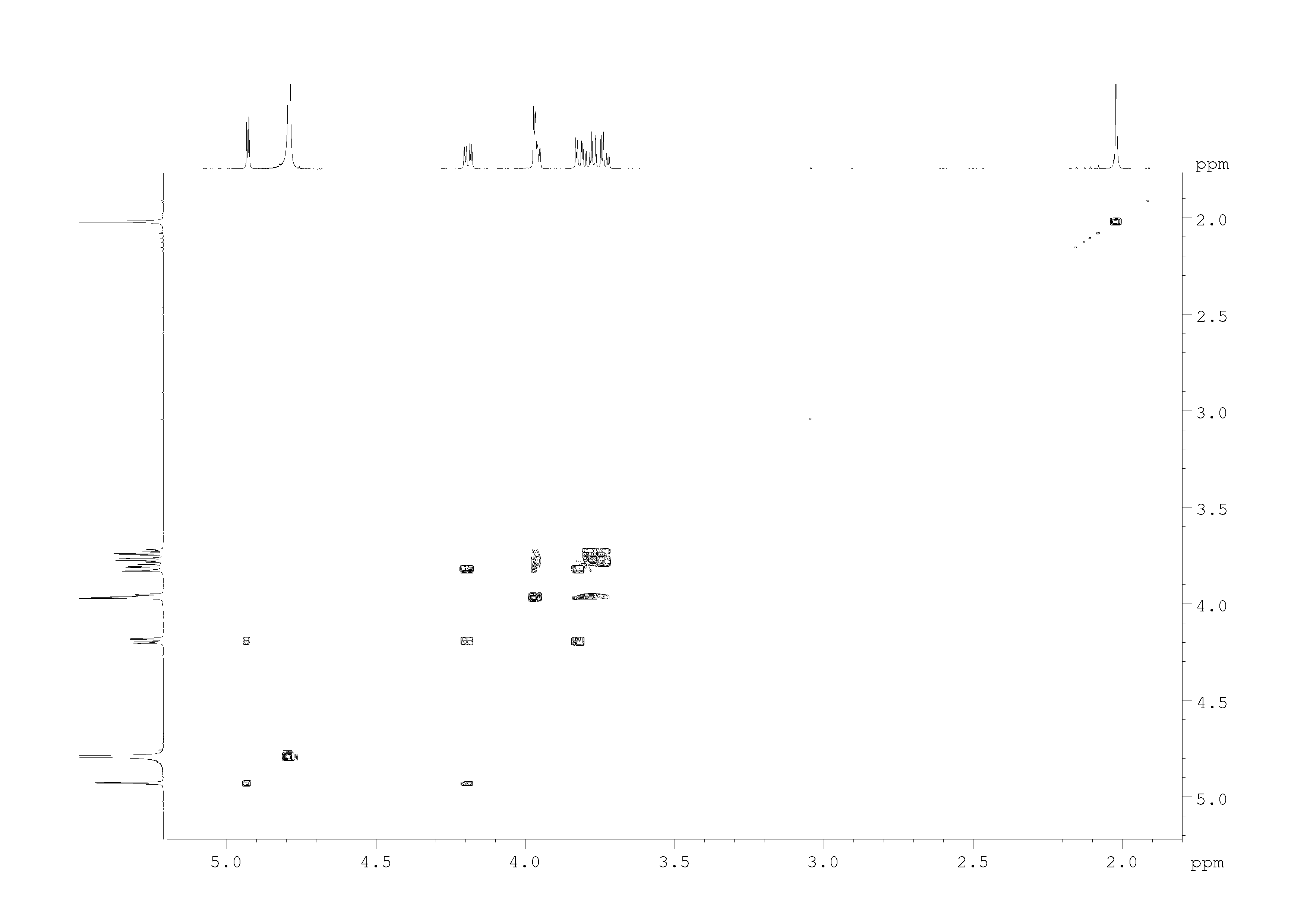
***2D-COSY NMR Spectra of Tn-ONH_2_ (**2**).

***
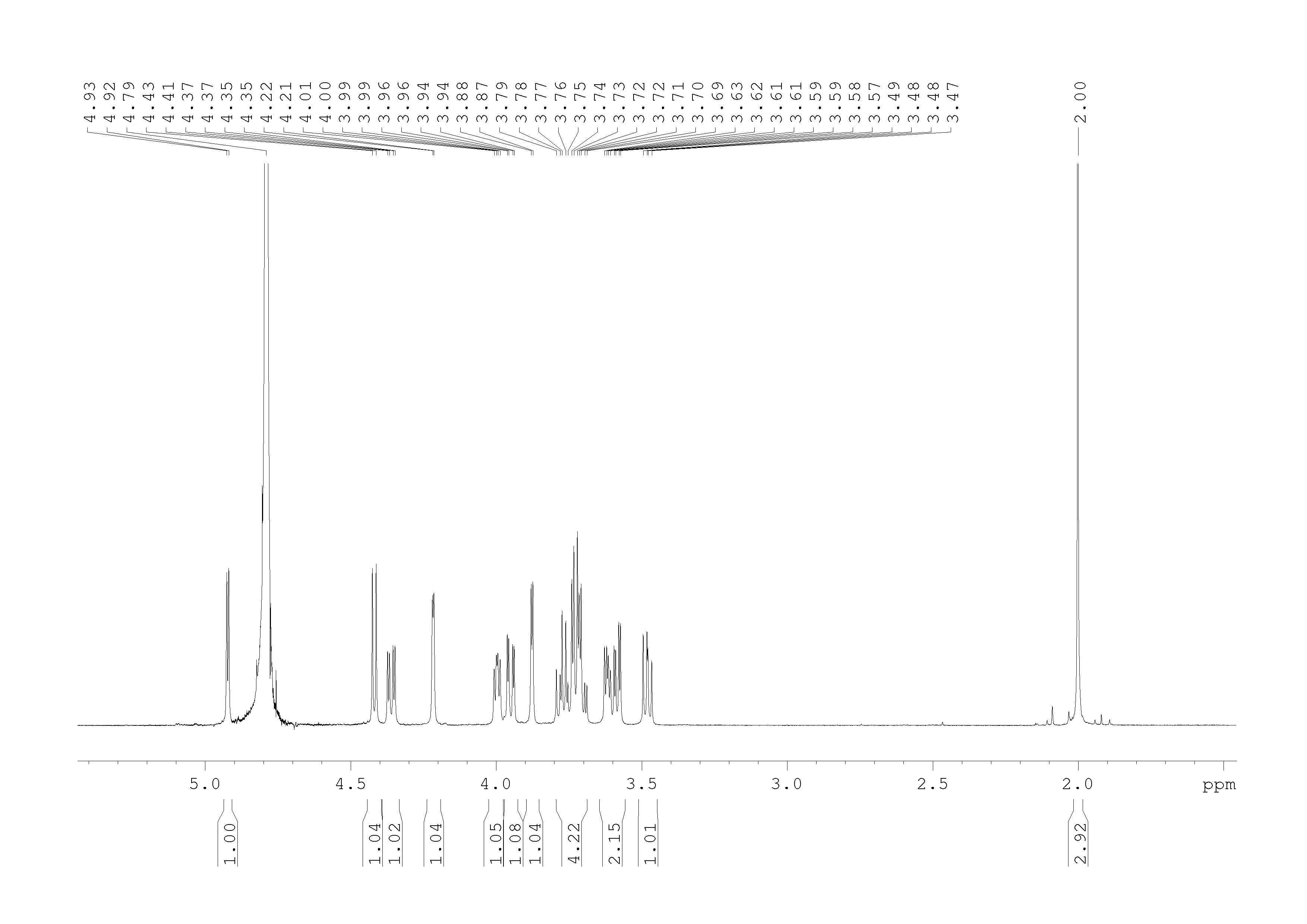
***^1^H NMR Spectra of TF-ONH_2_ (**3**).

***
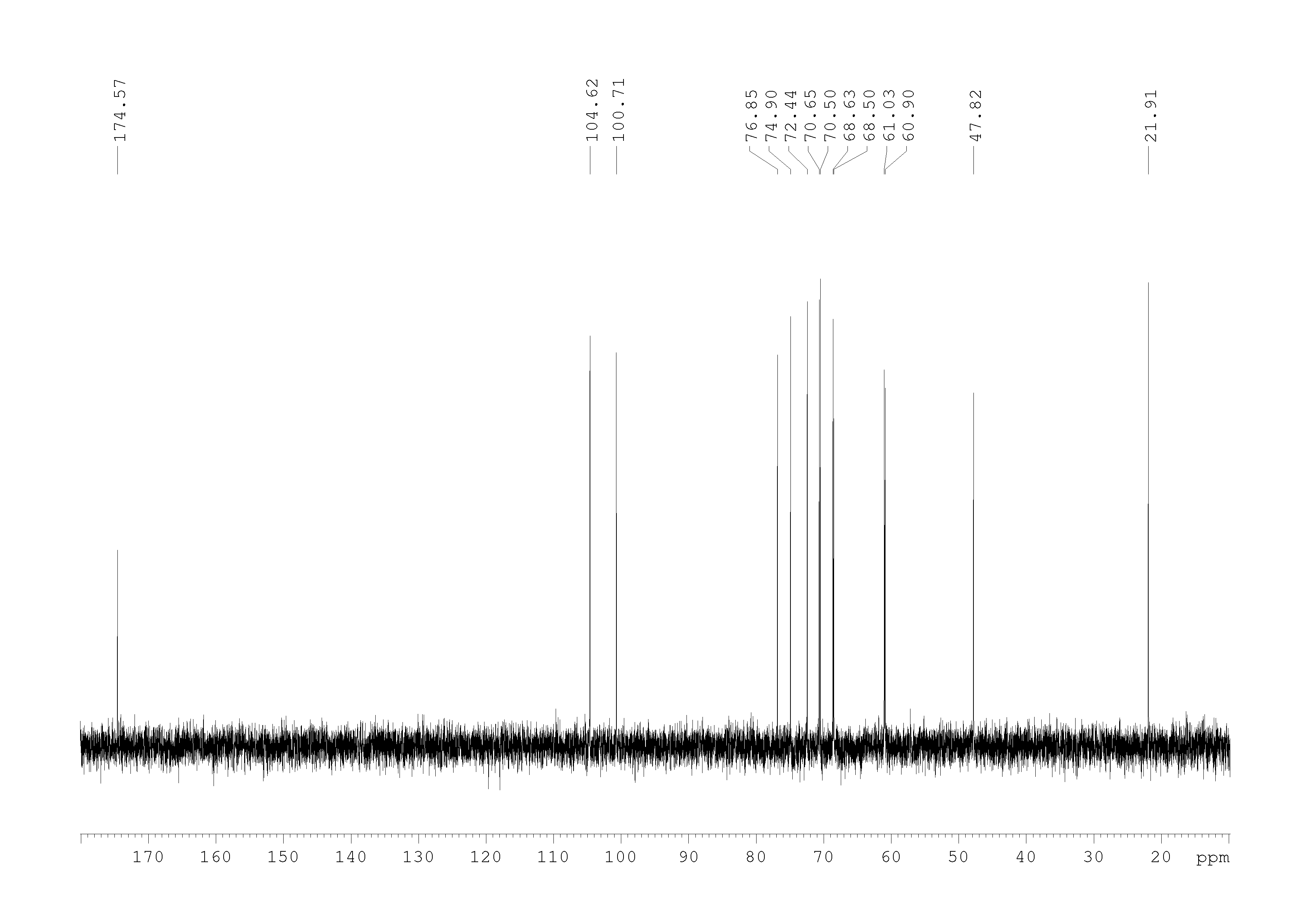
***^13^C NMR Spectra of TF-ONH_2_ (**3**).

***
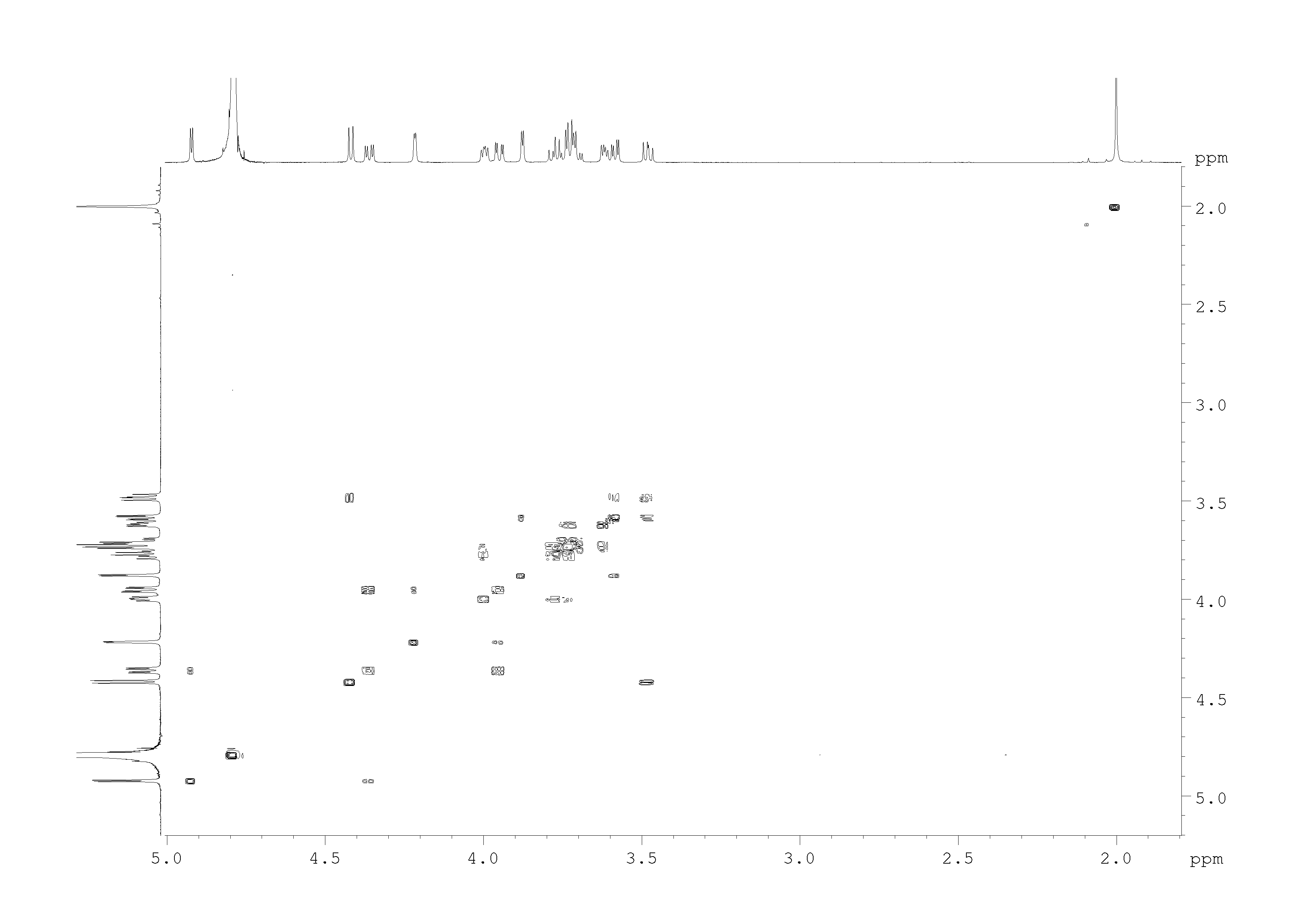
***2D-COSY NMR Spectra of TF-ONH_2_ (**3**).

*
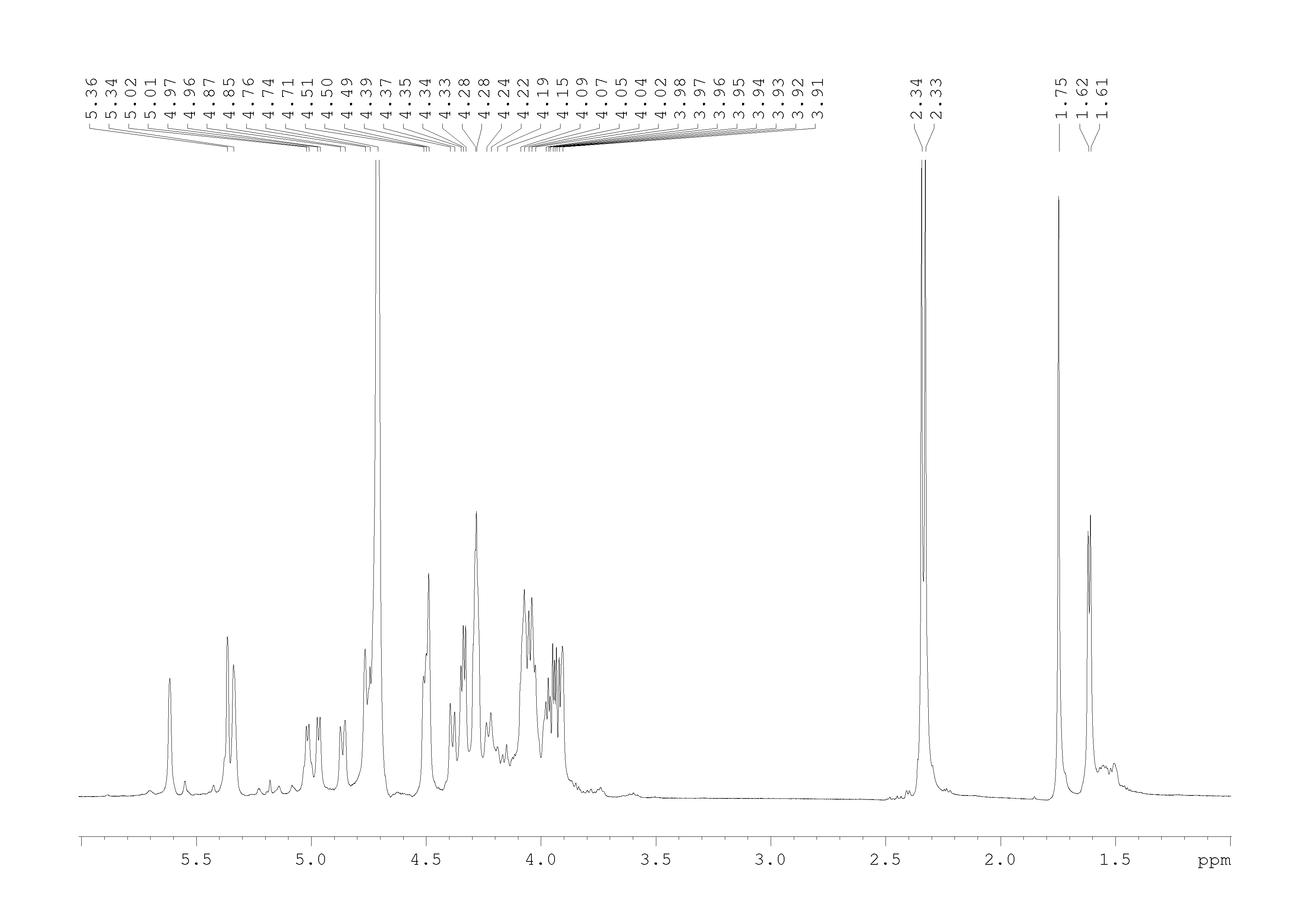
*^1^H NMR Spectra of PS A1 (**1**).

***
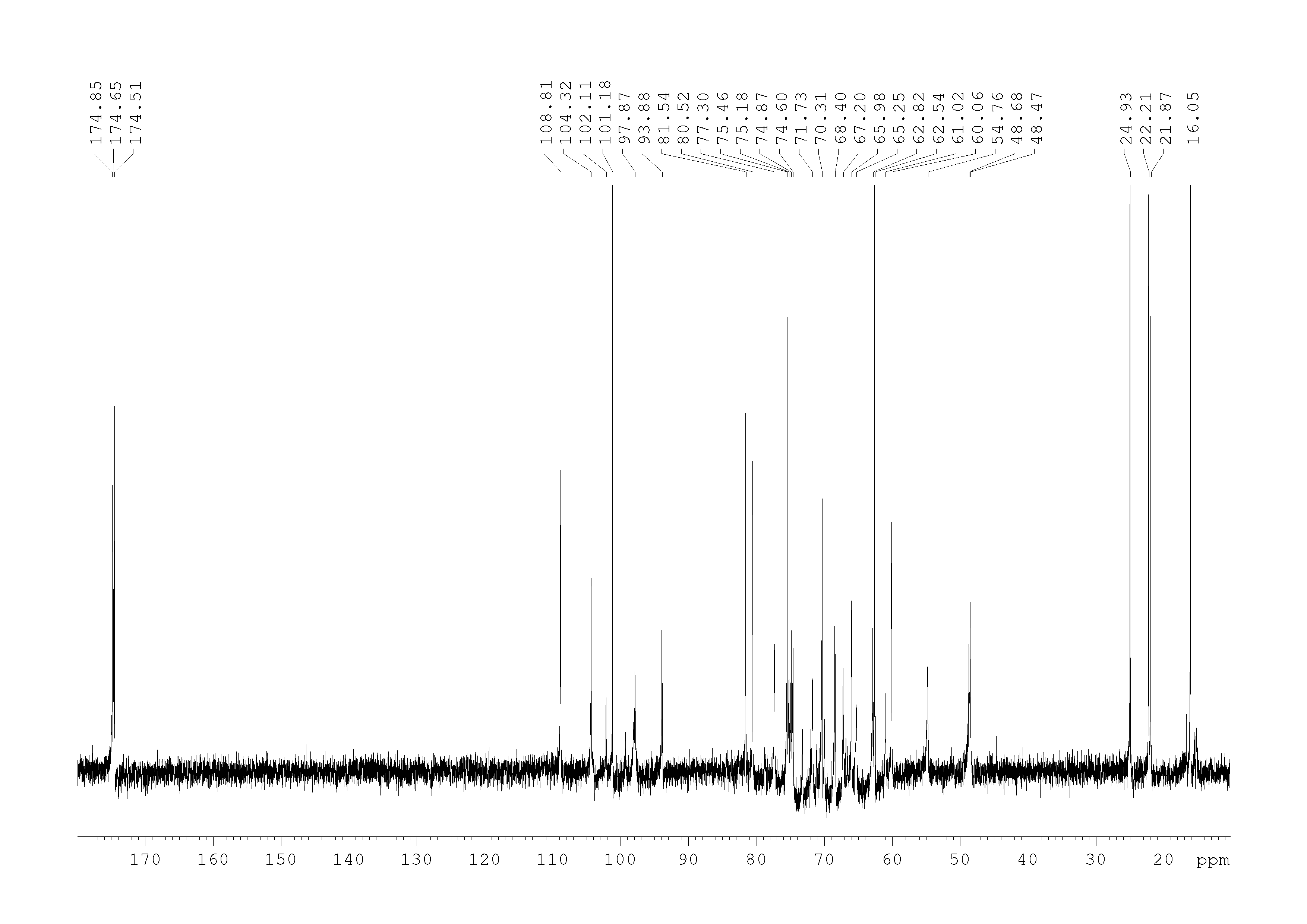
***^13^C NMR Spectra of PS A1 (**1**).

***
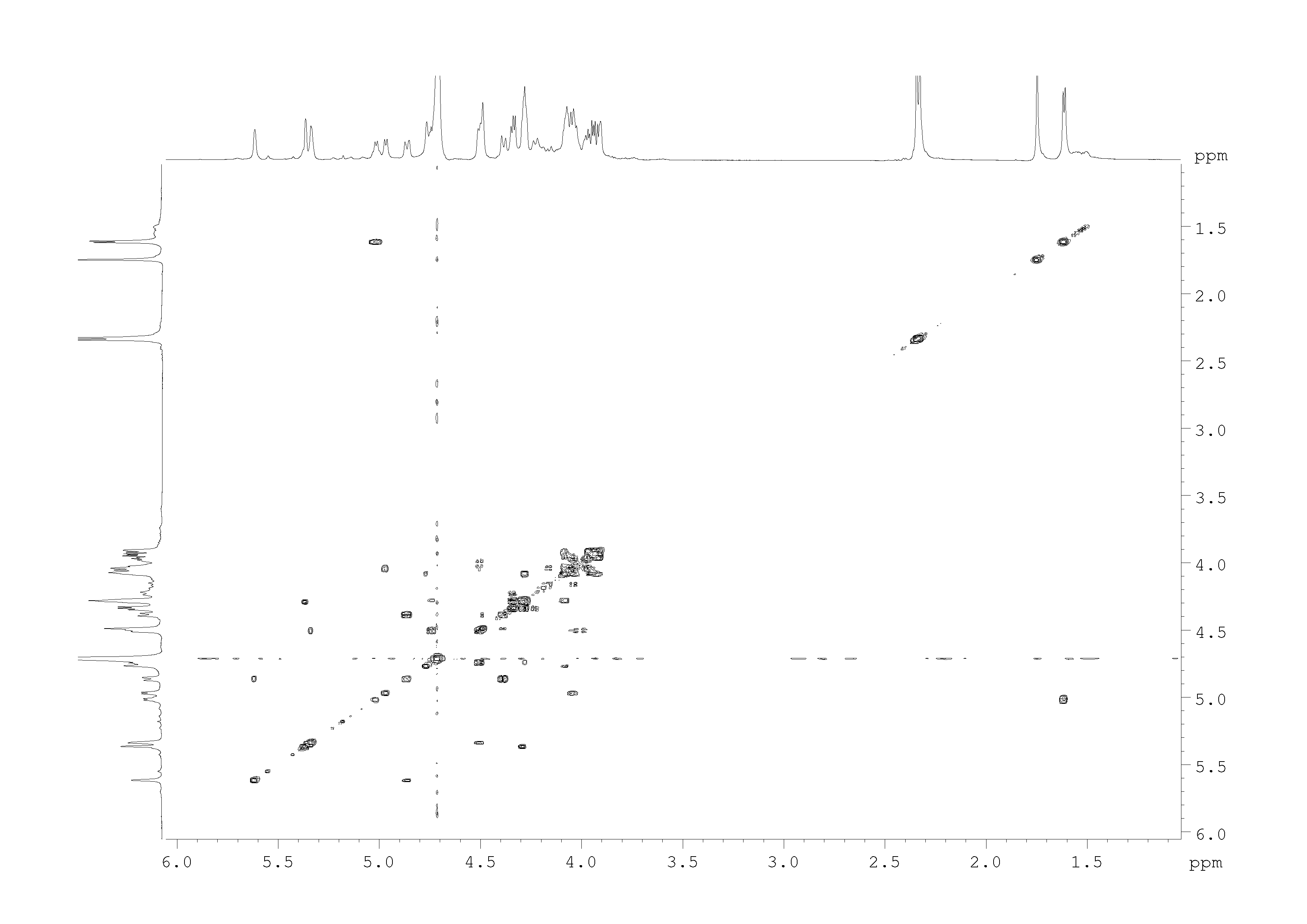
***2D-COSY NMR Spectra of PS A1 (**1**).

***
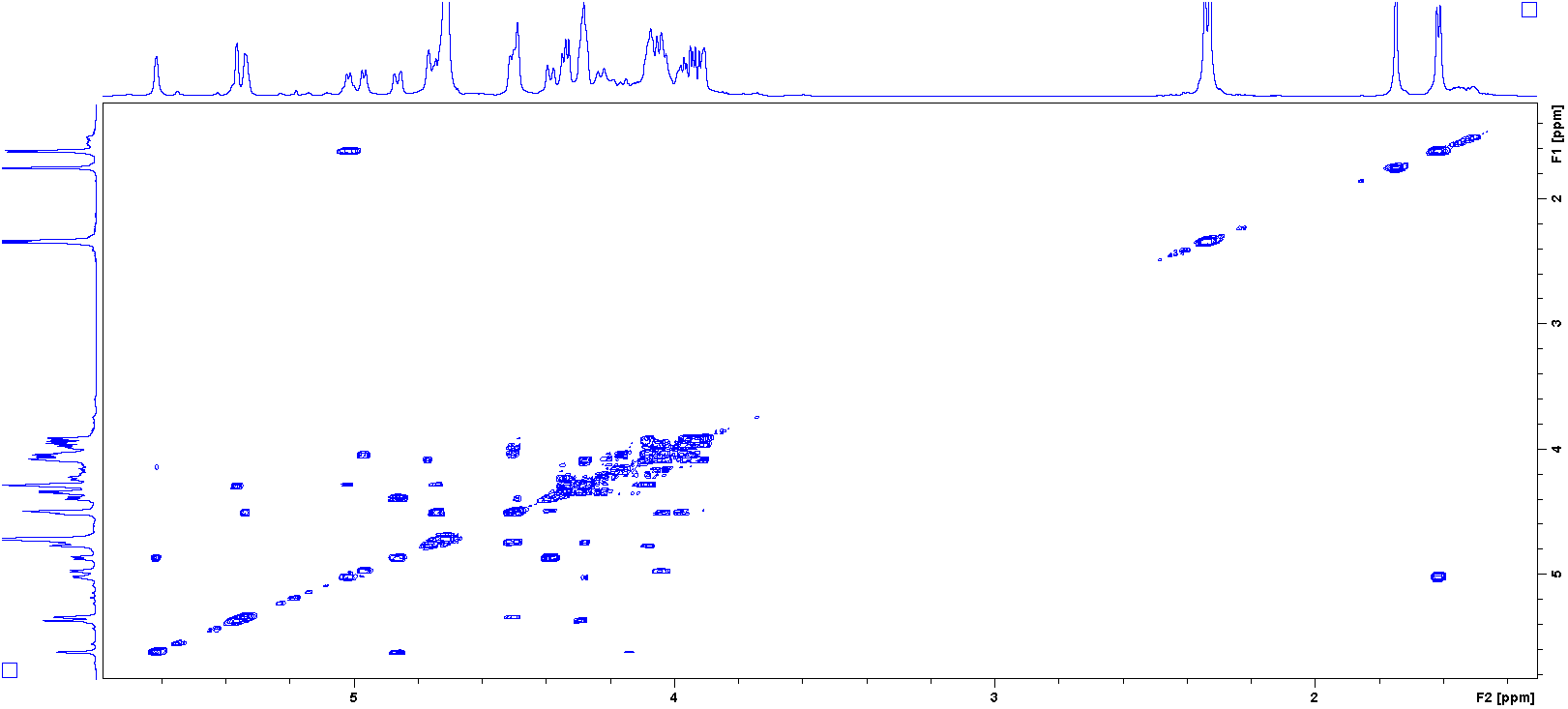
***

1D-TOCSY of PS A1 (**1**) constituent spin systems.


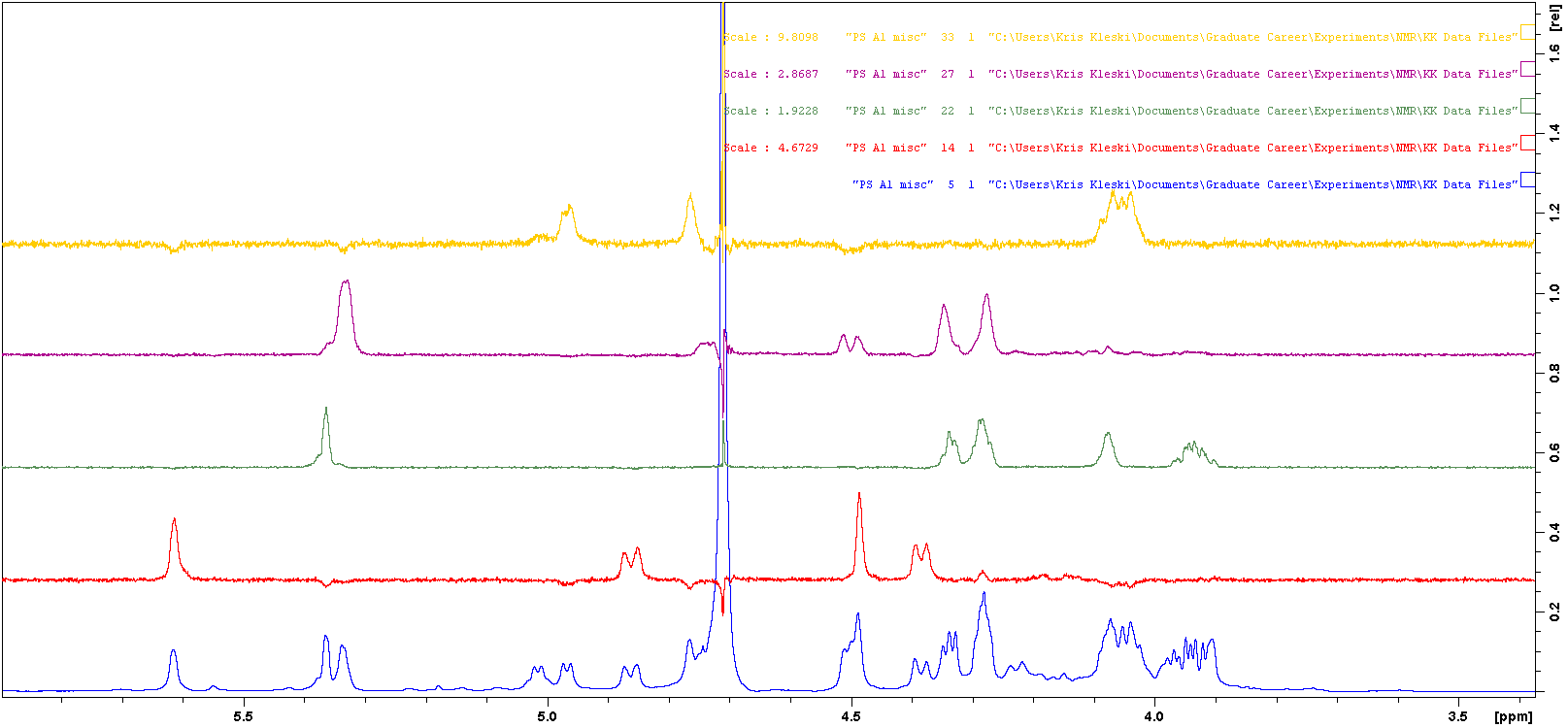


***
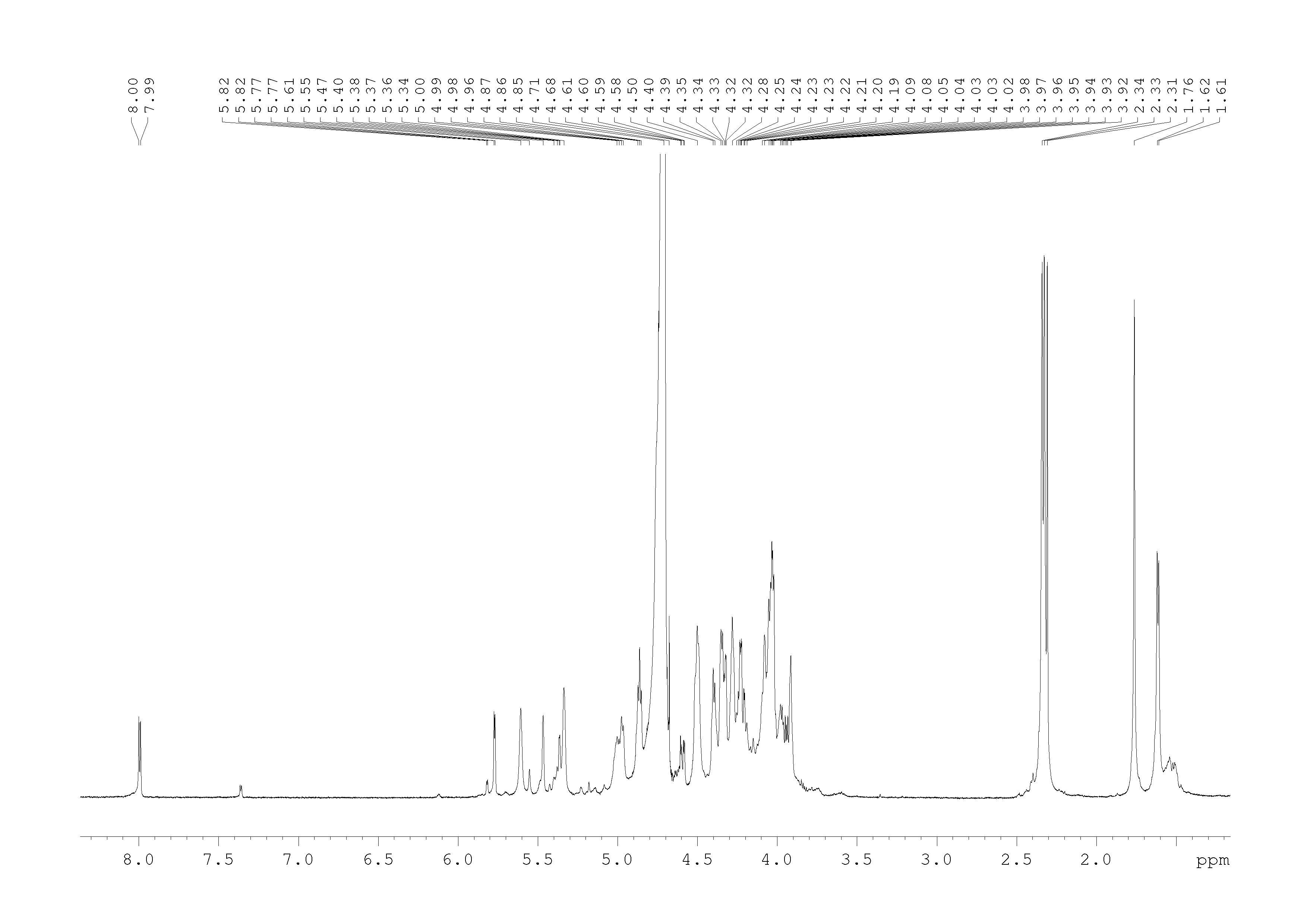
***^1^H NMR Spectra of Tn-PS A1 (**4a**).

***
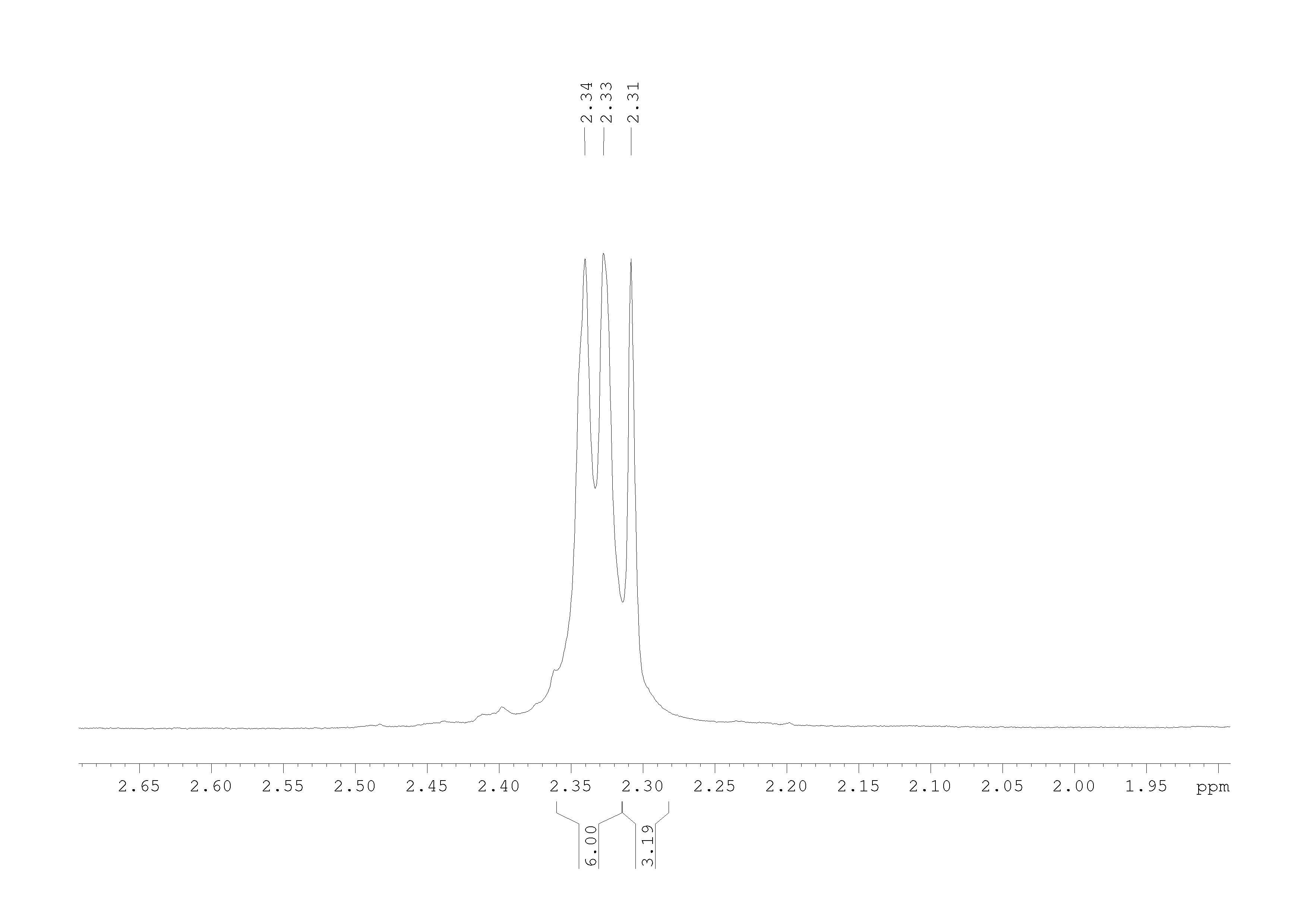
***^1^H NMR Spectra of Tn-PS A1 (**4a**; -NHAc Integrals).

***
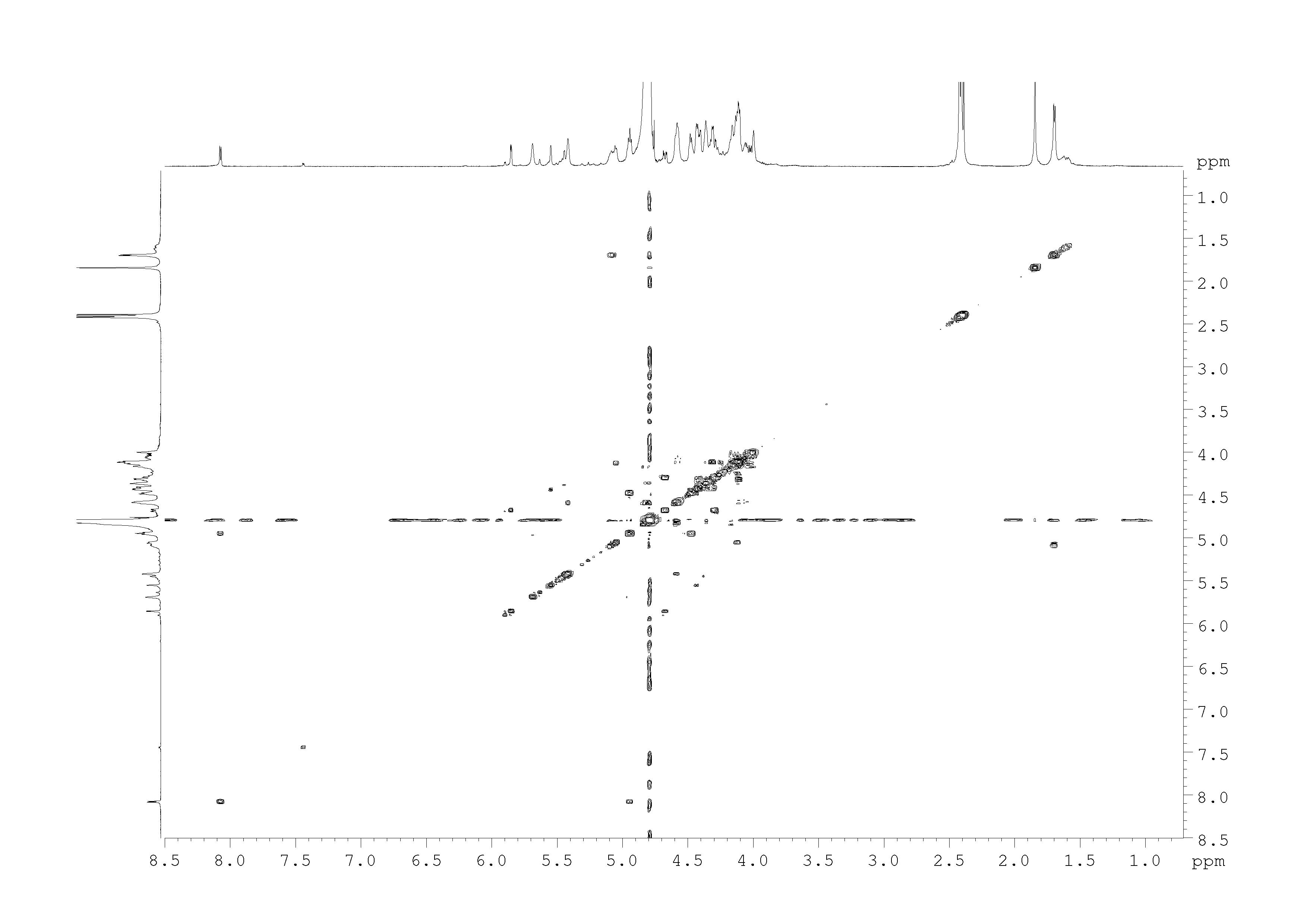
***2D-COSY NMR Spectra of Tn-PS A1 (**4a**).

**

***
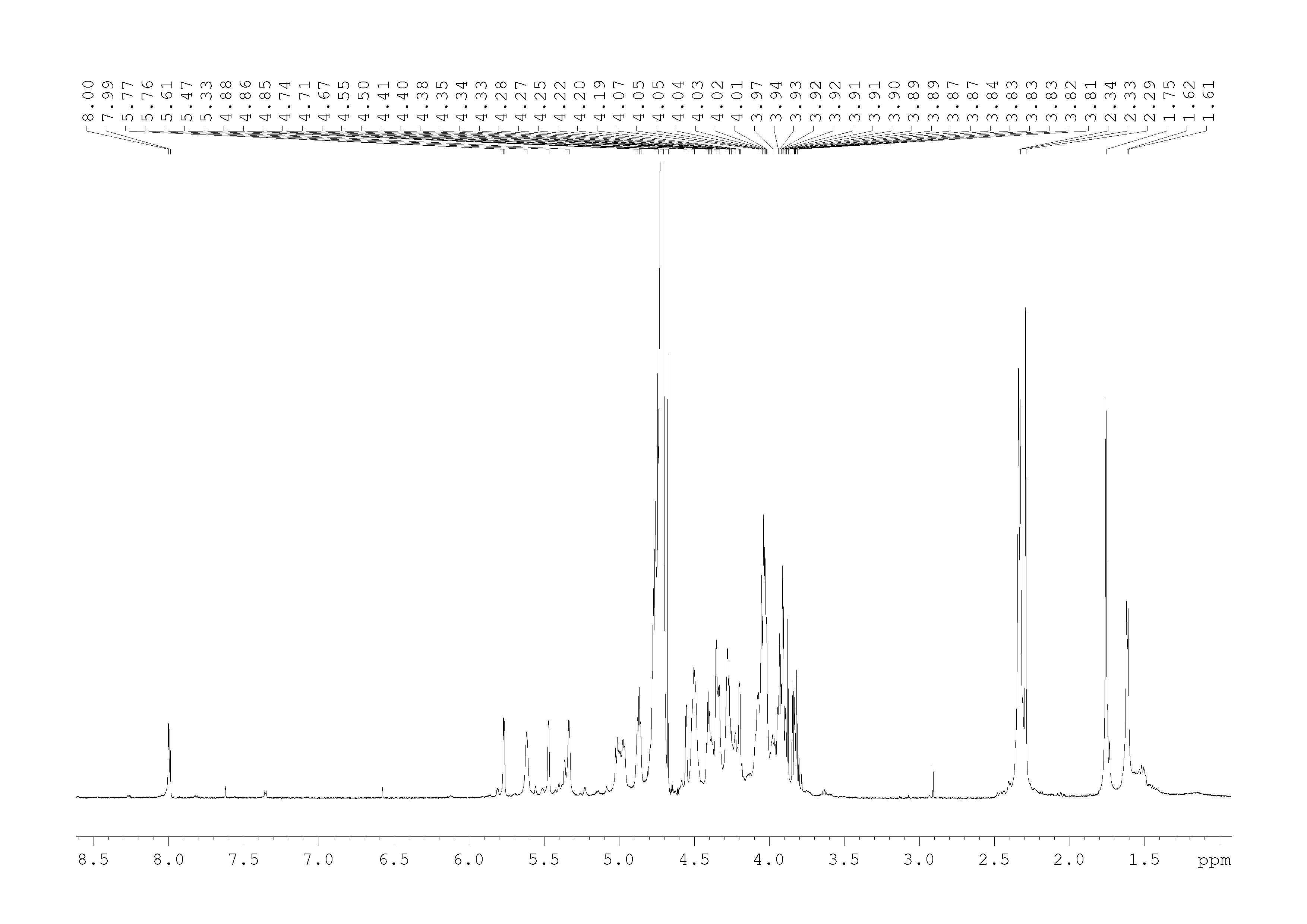
***^1^H NMR Spectra of TF-PS A1 (**4b**).

***
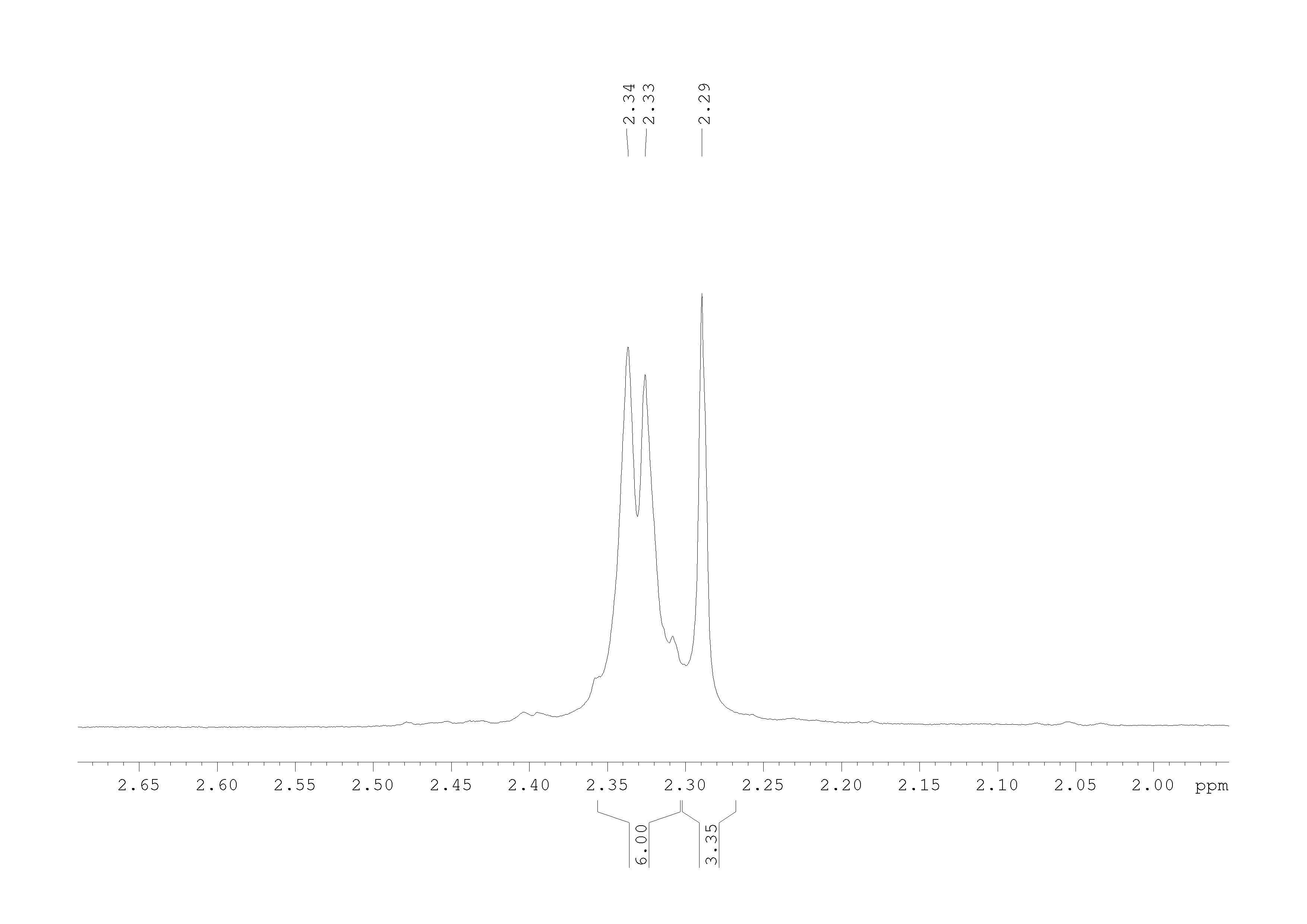
***^1^H NMR Spectra of TF-PS A1 (**4b**; -NHAc Integrals)

******

***
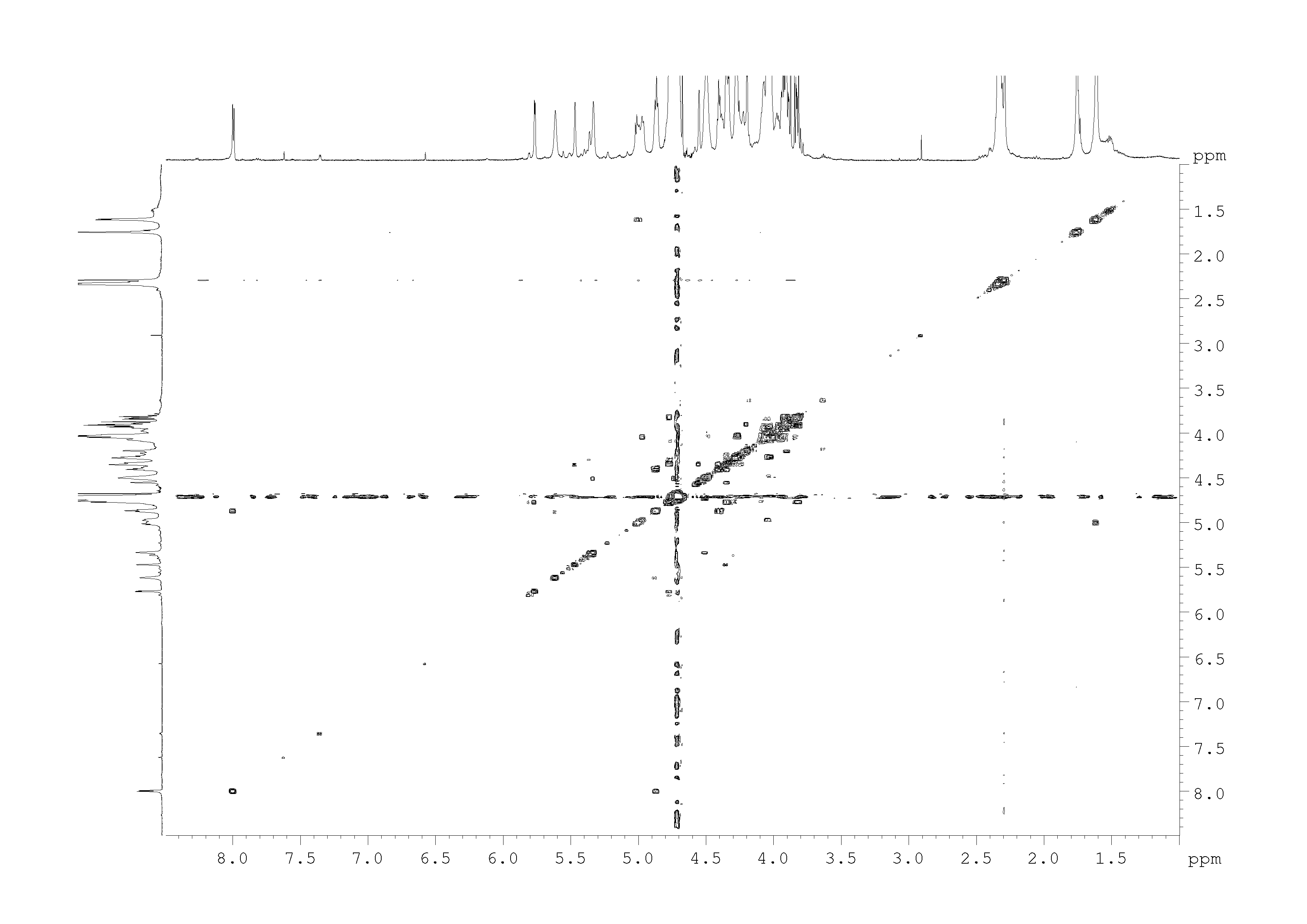
***2D-COSY NMR Spectra of TF-PS A1 (**4b**).

******

***
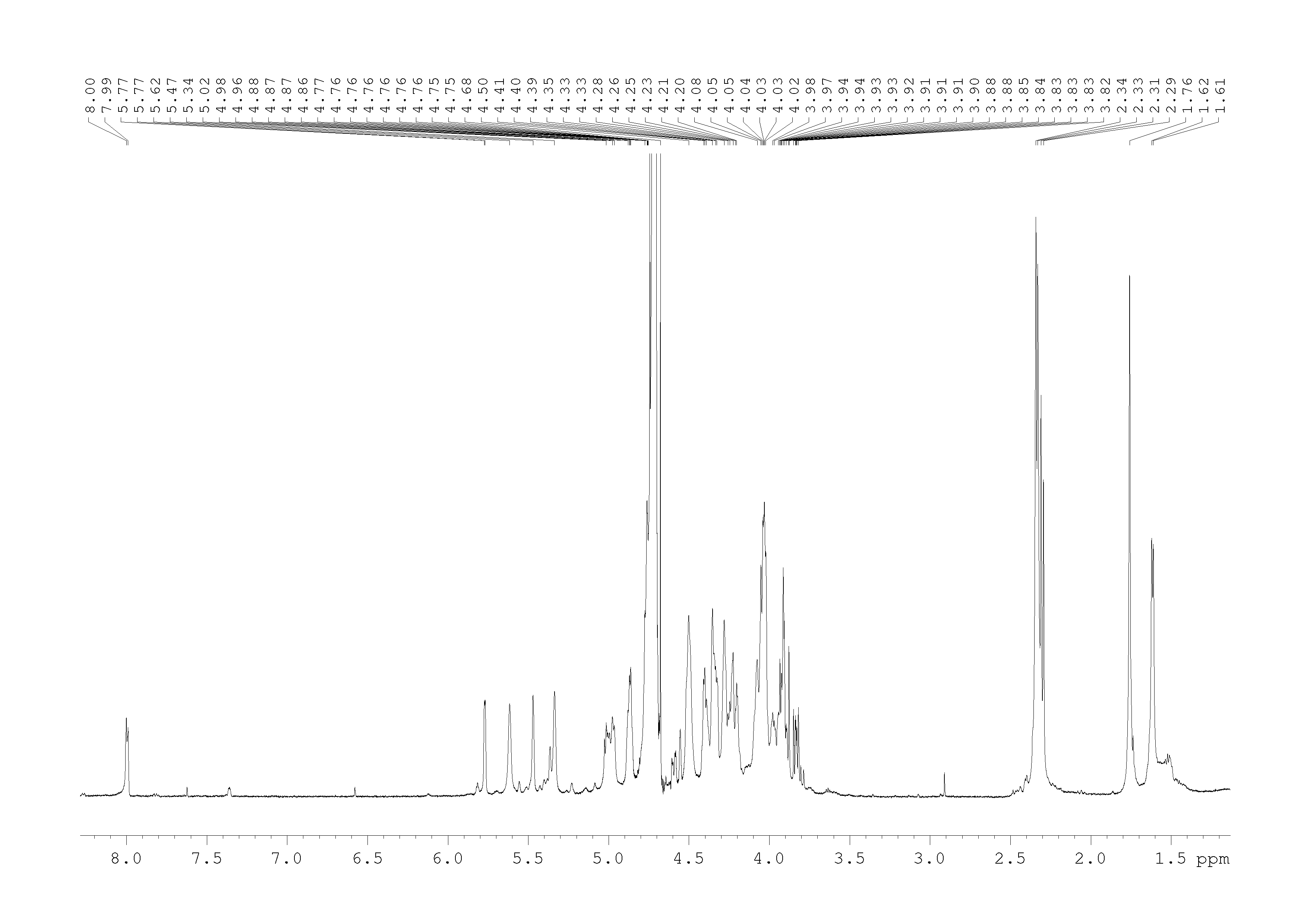
***^1^H NMR Spectra of Tn-TF-PS A1 (**4c**).

***
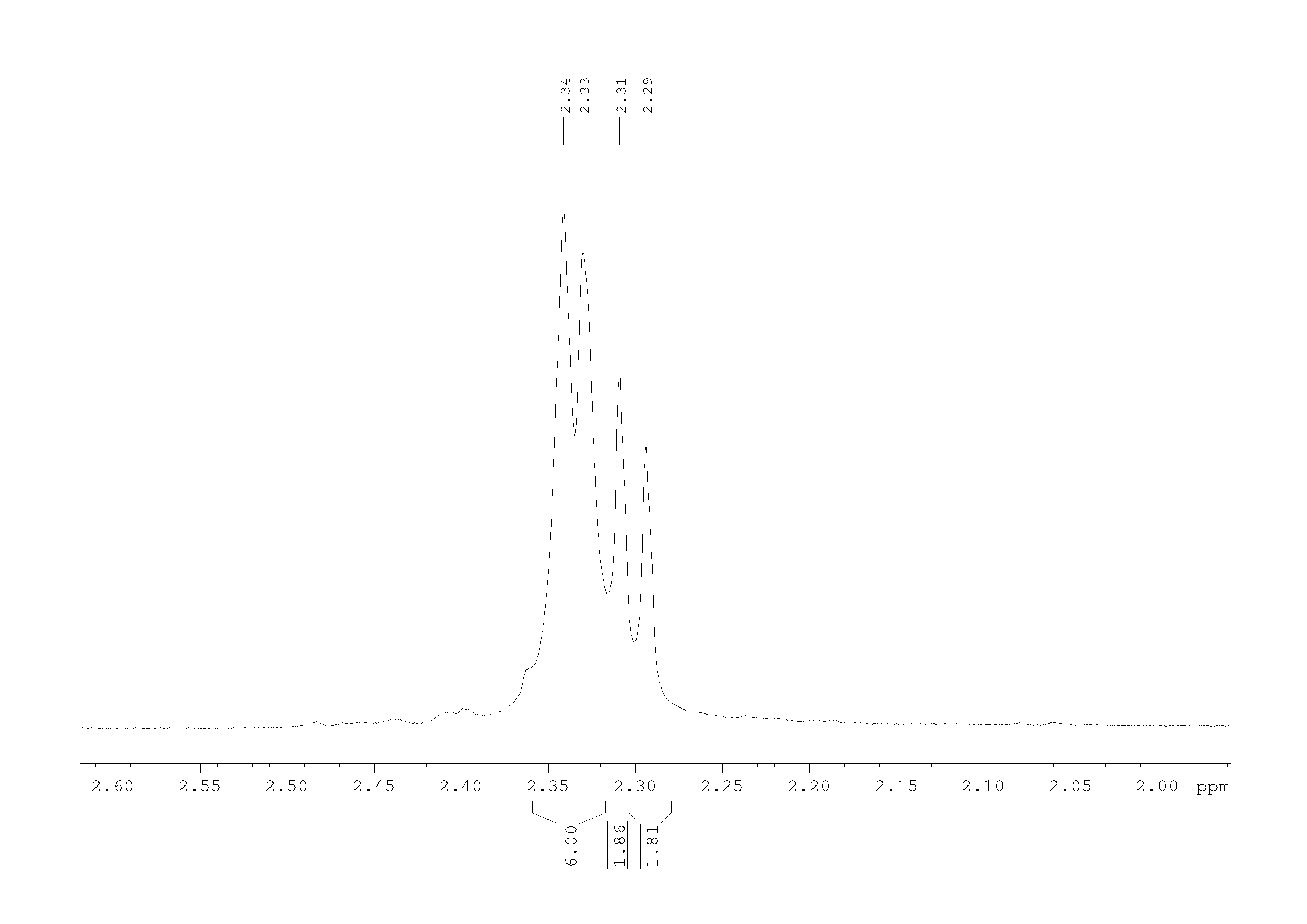
***^1^H NMR Spectra of Tn-TF-PS A1 (**4c**; -NHAc Integrals)

******

***
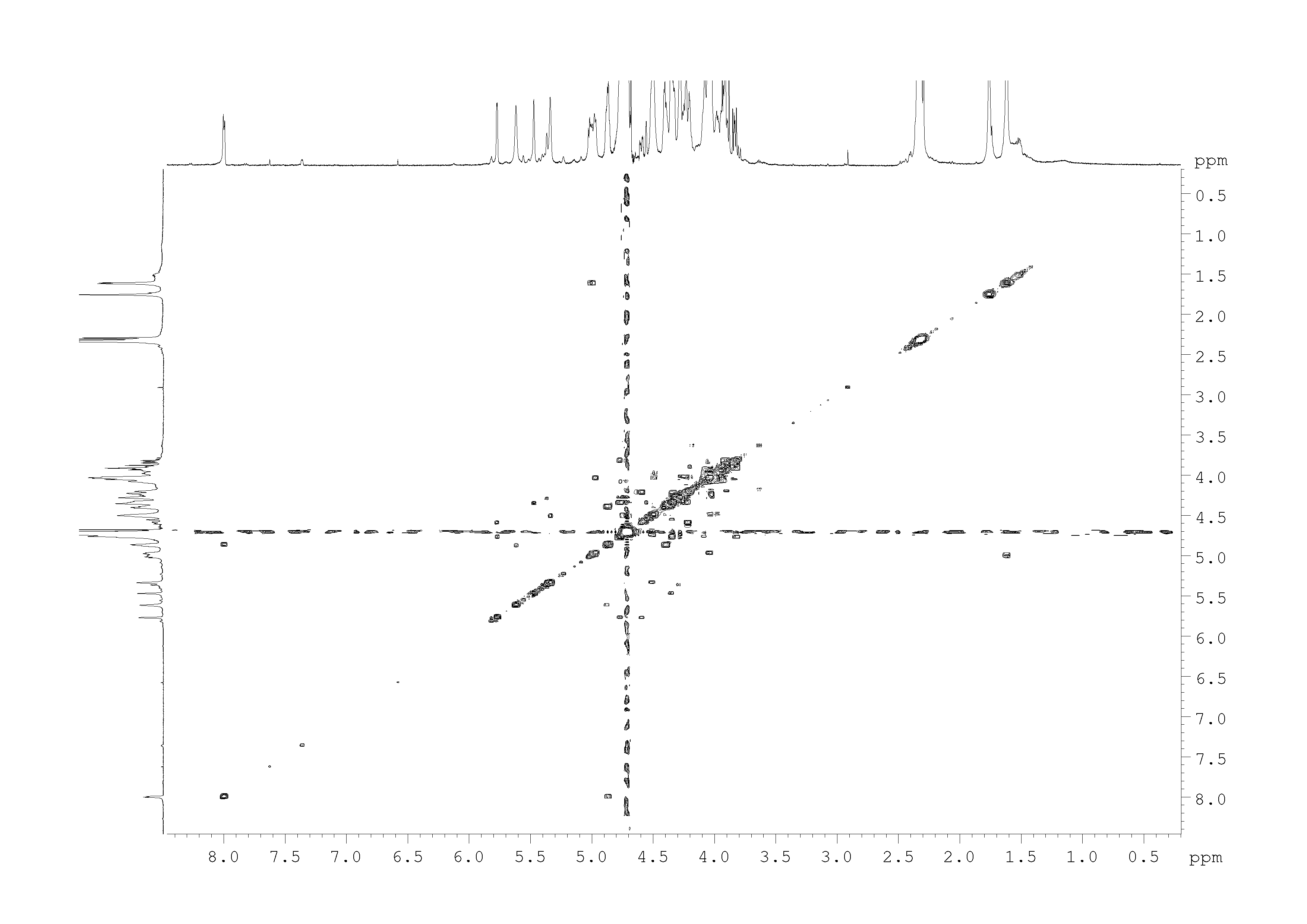
***2D-COSY NMR Spectra of Tn-TF-PS A1 (**4c**).

******

***
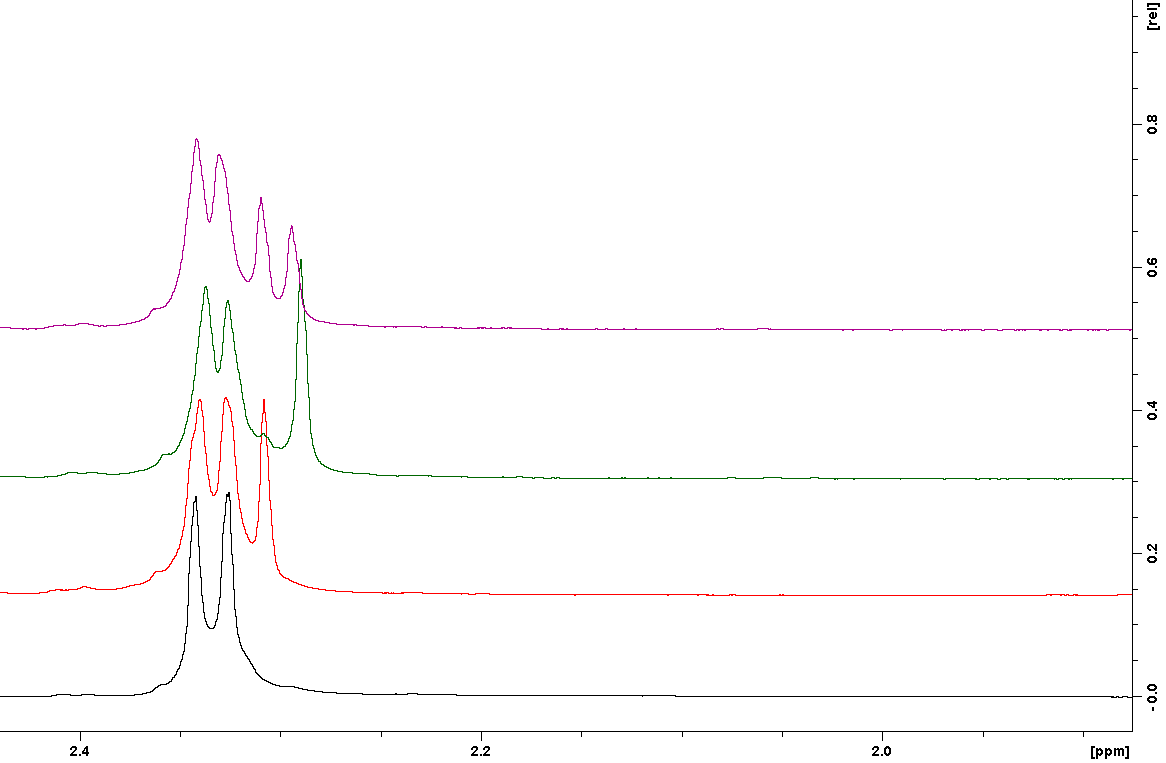
***Comparison of overlaying ^1^H spectra from **1** and **4a-c**.

**PS A1 (1)**

**Tn-PS A1 (4a)**

**TF-PS A1 (4b)**

**Tn-TF-PS A1 (4c)**

1D-TOCSY of Tn-TF-PS A1 (**4c**) showing doublet of (Tn-TF-GalNac) at 5.78 ppm at 60 ^o^C with 120 ms mixing time. Both GalNac, either from Tn or TF, were indistinguishable due to overlap.

***
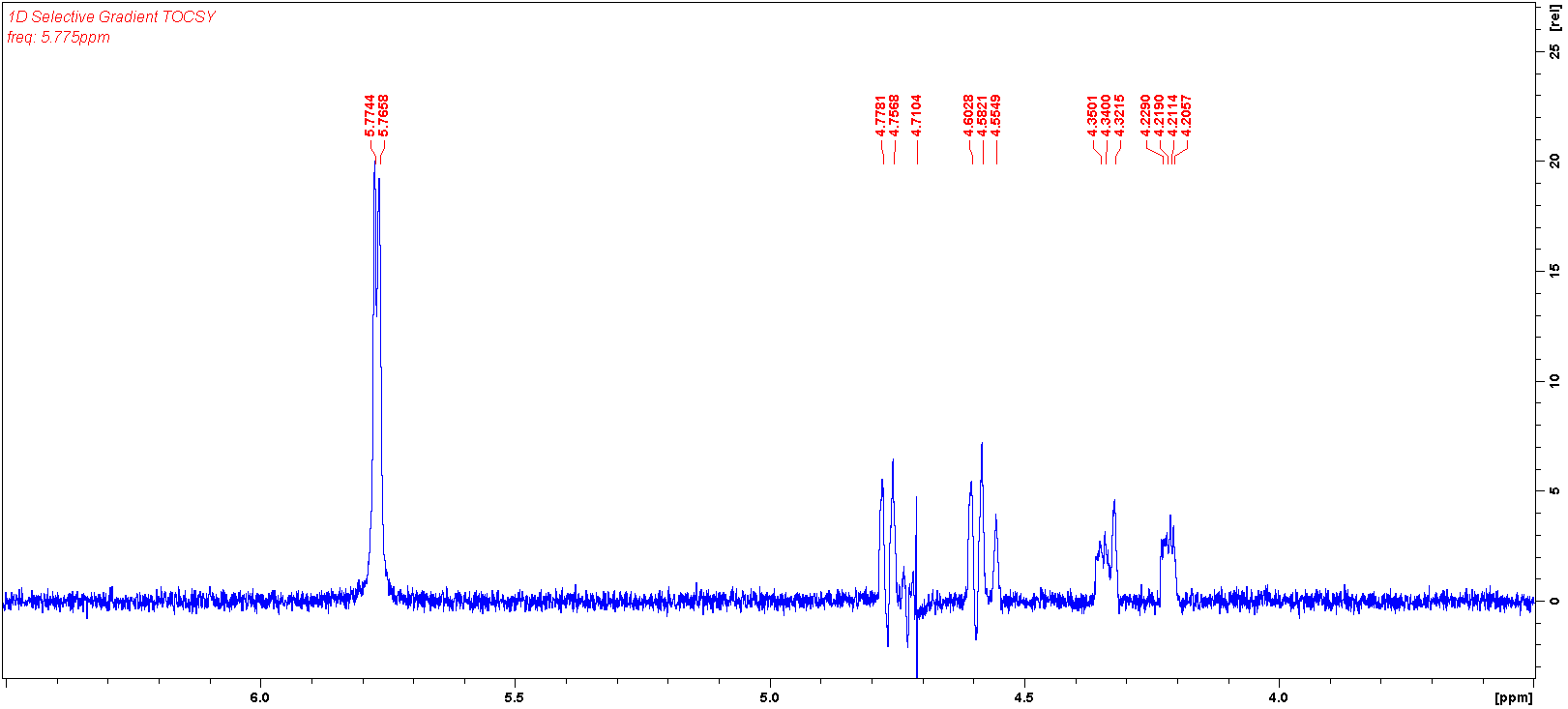
***

1D-TOCSY of Tn-TF-PS A1 (**4c**) showing doublet of (TF-Gal) at 4.77 ppm at room temperature with 120 ms mixing time.

***
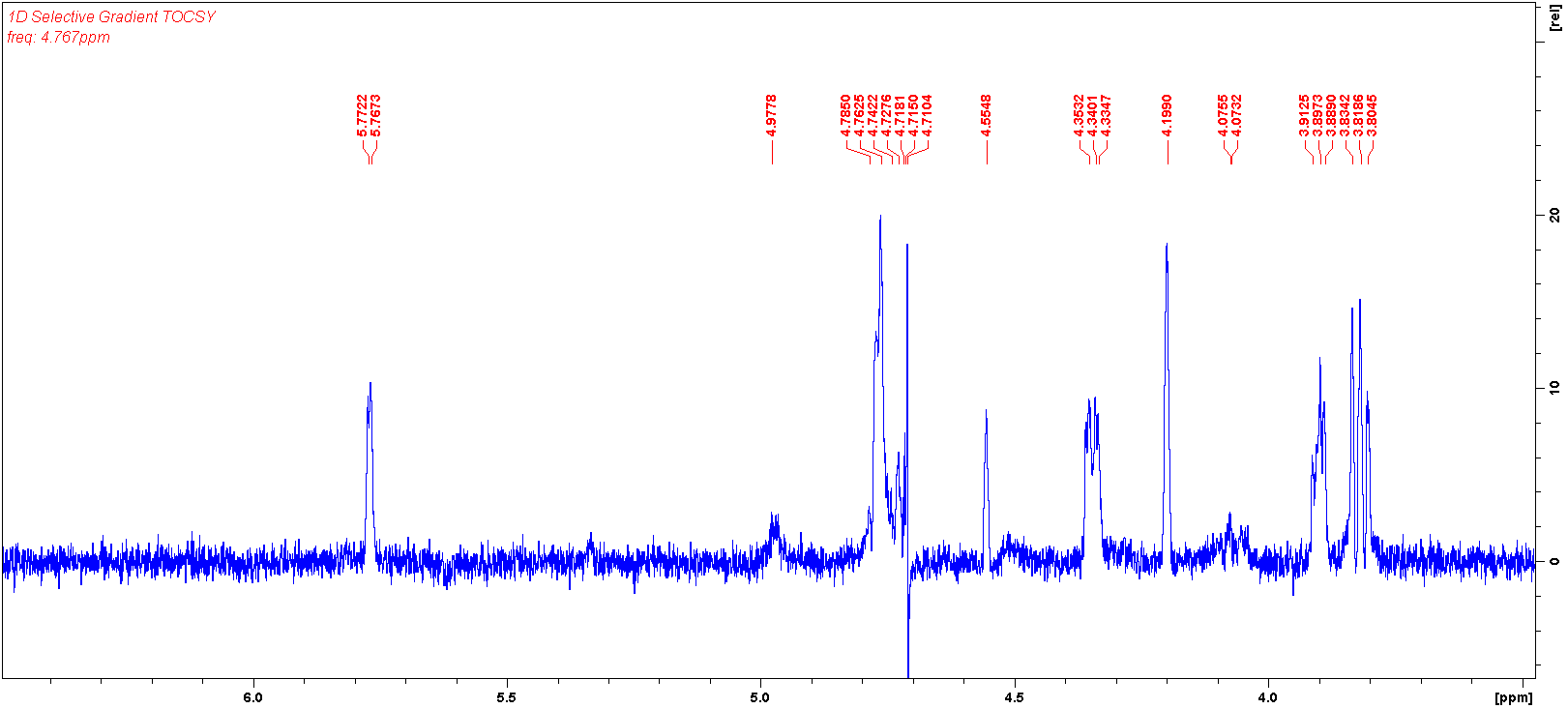
***
