# Supplementary material for: Enhanced Immune Response Against the Thomsen-Friedenreich Tumor Antigen Using a Bivalent Entirely Carbohydrate Conjugate
Source: Molecules. 2020 Mar 13;25(6):1319. doi: 10.3390/molecules25061319 (PMC7144725; doi:10.3390/molecules25061319)
Supplement: Supplementary file 1 [file molecules-25-01319-s001.zip › SI/Doc. S2-Table S1.docx]

**Table S1.** Percent loading calculation and formulas:

Tn: $\% Loading=\frac{MW Tn-ONH_{2}}{MW Tn-PS A1 repeating unit}*\frac{integral of NHAc methyl group from Tn}{integral of NHAc methyl group from PS A1}$

TF: $\% Loading=\frac{MW TF-ONH_{2}}{MW TF-PS A1 repeating unit}*\frac{integral of NHAc methyl group from TF}{integral of NHAc methyl group from PS A1}$

| **Name** | **Molecular Formula** | **Molecular Weight** | **Integral of PS A1 -NHAc** | **Integral of Hapten -NHAc** | **% Loading** |
| --- | --- | --- | --- | --- | --- |
| **Tn-ONH_2_ (2)** | **C_8_H_16_N_2_O_6_** | **236.2240** | **NA** | **NA** | **NA** |
| **TF-ONH_2_ (3)** | **C_14_H_26_N_2_O_11_** | **398.3650** | **NA** | **NA** | **NA** |
| **PS A1 (1)** | **C_33_H_55_N_3_O_20_** | **813.8040** | **NA** | **NA** | **NA** |
| **Tn-PS A1 (4a)** | **C_40_H_65_N_5_O_24_** | **999.9710** | **6.00** | **3.19** | **12.6** |
| **TF-PS A1 (4b)** | **C_46_H_75_N_5_O_29_** | **1162.1120** | **6.00** | **3.35** | **19.1** |
| **Tn-TF-PS A1 (4c)** | **NA** | **NA** | **6.00** | **1.85 (Tn): 1.80 (TF)** | **7.28(Tn): 10.3 (TF)** |
