# Supplementary material for: Enhanced Immune Response Against the Thomsen-Friedenreich Tumor Antigen Using a Bivalent Entirely Carbohydrate Conjugate
Source: Molecules. 2020 Mar 13;25(6):1319. doi: 10.3390/molecules25061319 (PMC7144725; doi:10.3390/molecules25061319)
Supplement: Supplementary file 1 [file molecules-25-01319-s001.zip › SI/Doc. S3-SchemeS1-S2.docx]

**Scheme S1.** General scheme for TACA-BSA conjugates.

**Scheme S2.** Syntheses of biotinylated TACA-PS A1 (**5a-c**) from TACA-conjugates (**4a-c**) as MGL2 assay probes.
