# Supplementary material for: Enhanced Immune Response Against the Thomsen-Friedenreich Tumor Antigen Using a Bivalent Entirely Carbohydrate Conjugate
Source: Molecules. 2020 Mar 13;25(6):1319. doi: 10.3390/molecules25061319 (PMC7144725; doi:10.3390/molecules25061319)
Supplement: Supplementary file 1 [file molecules-25-01319-s001.zip › SI/Doc. S4-FigS1-S6.docx]

**Figure S1.** Individual mouse titer values obtained from immunization with Tn-PS A1, TF-PS A1, or Tn-TF-PS A1 in conjunction with SAS against Tn-BSA or TF-BSA.

**
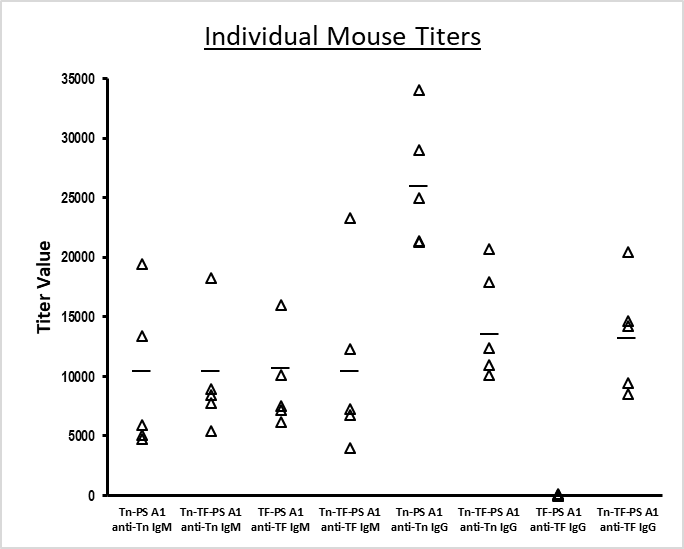
**

**Figure S2.** DLS experiments with PS A1 (**1**) and TACA-PS A1 (**4a-c**) constructs.

**
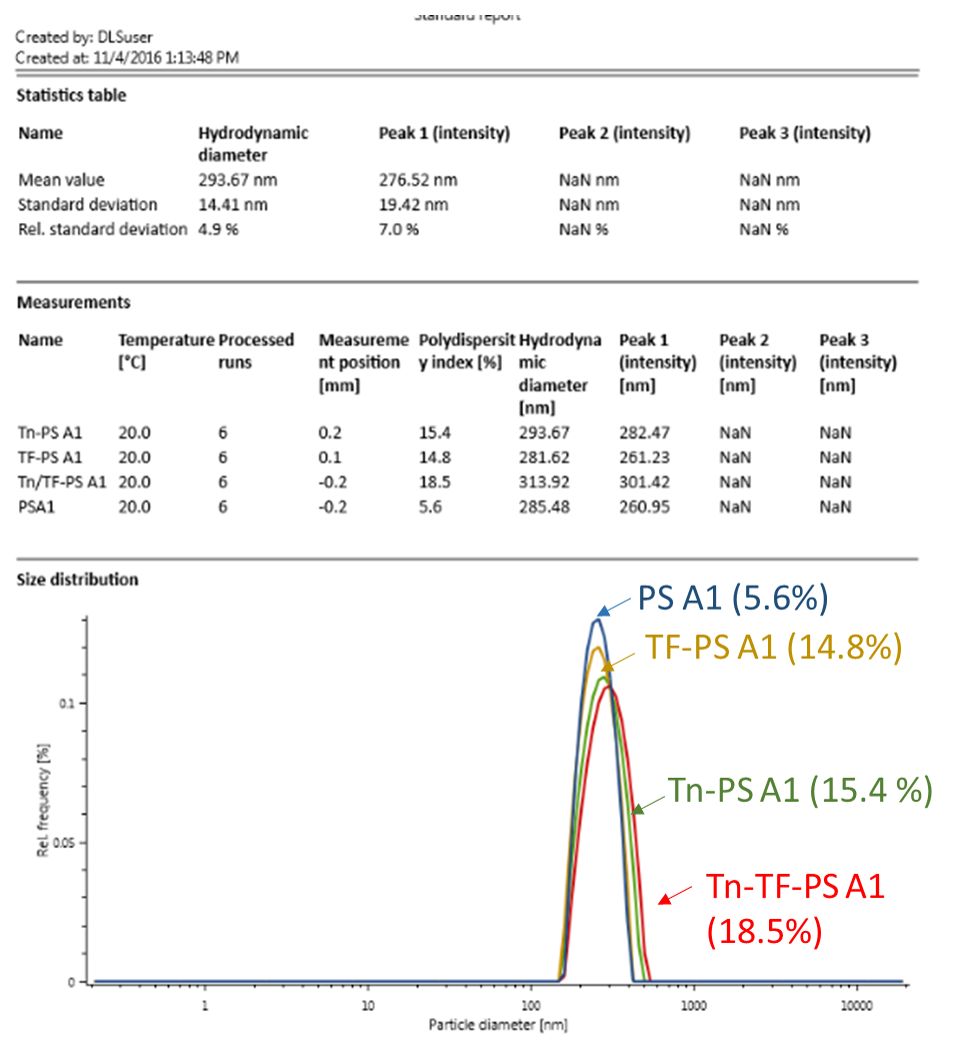
**

**Figure S3.** Flow cytometry with antiserum from PS A1 (**1**), TACA-PS A1 conjugates (**4a-c**), and PBS control using secondary Alexa Fluor® 488 anti-IgG and MCF-10A cells. These data show negligible binding of experimental antiserum to the healthy epithelial breast cell line MCF-10A.


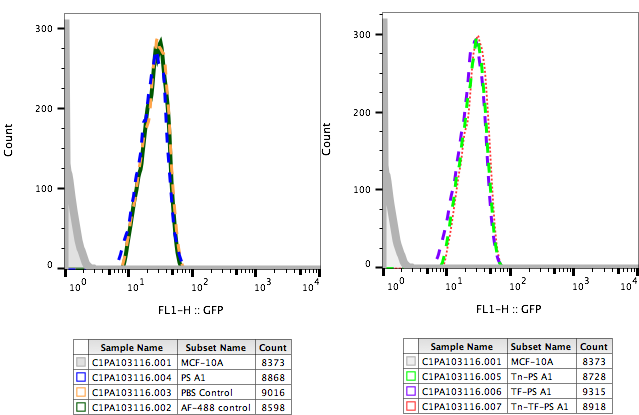


**Figure S4.** Representative pictures of IL-17 ELISpot followed by comprehensive bar chart of IL-17A ELISpot.

**
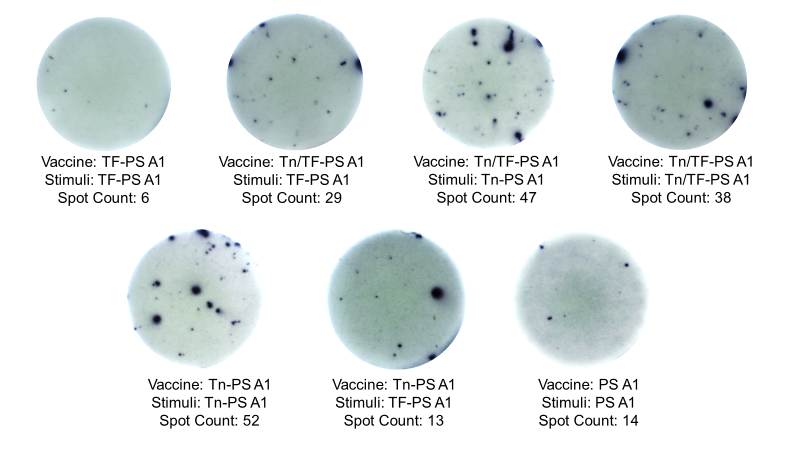
**

**
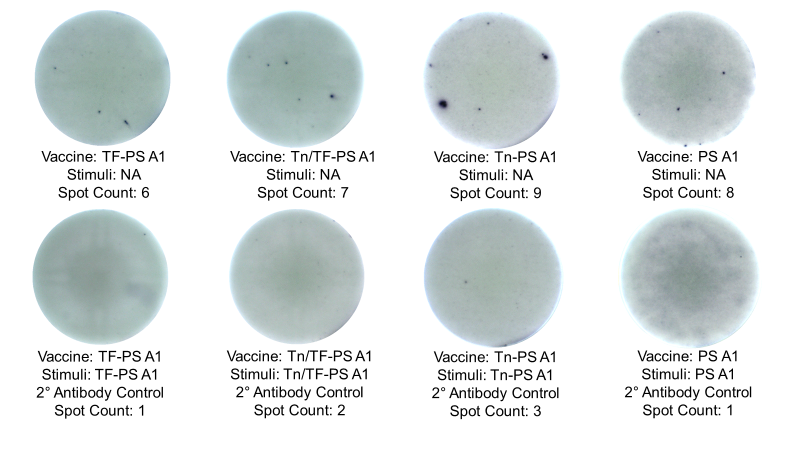
**

**Figure S5.** Representative pictures of IFN-γ ELISpot followed by a bar chart.

**
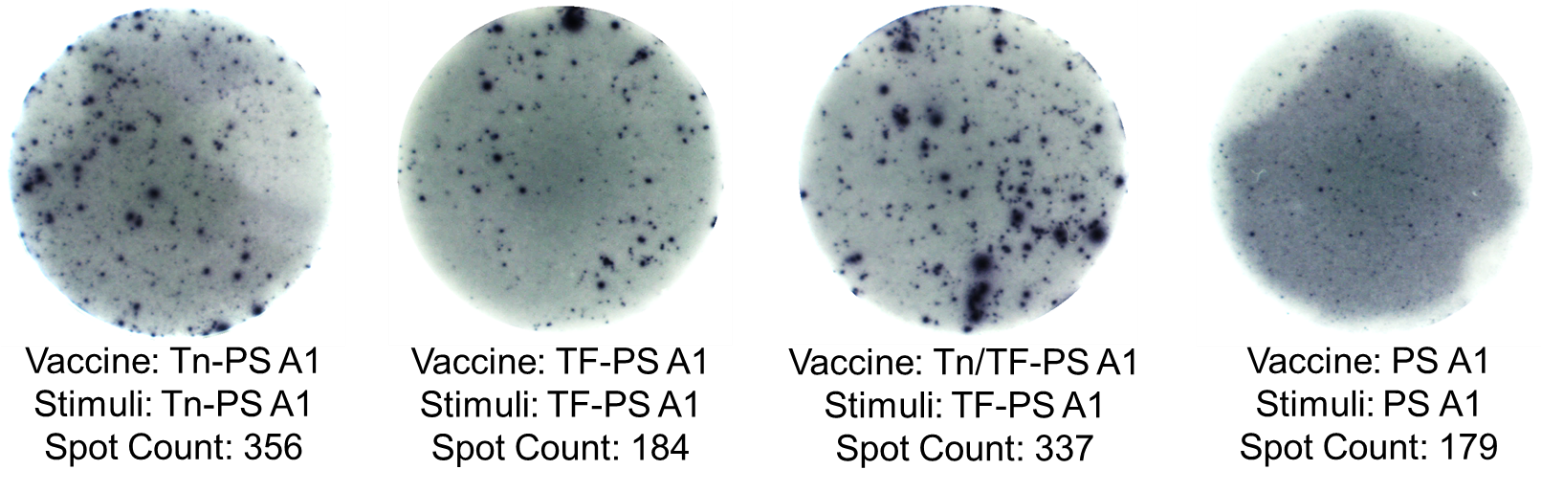
**

**
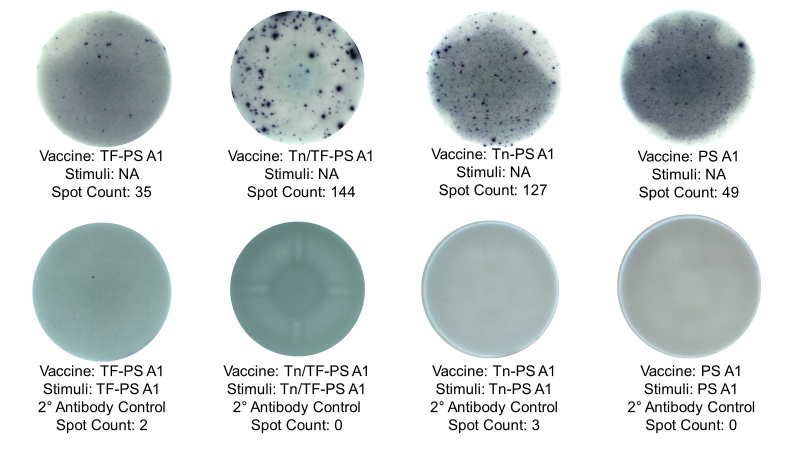
**

**
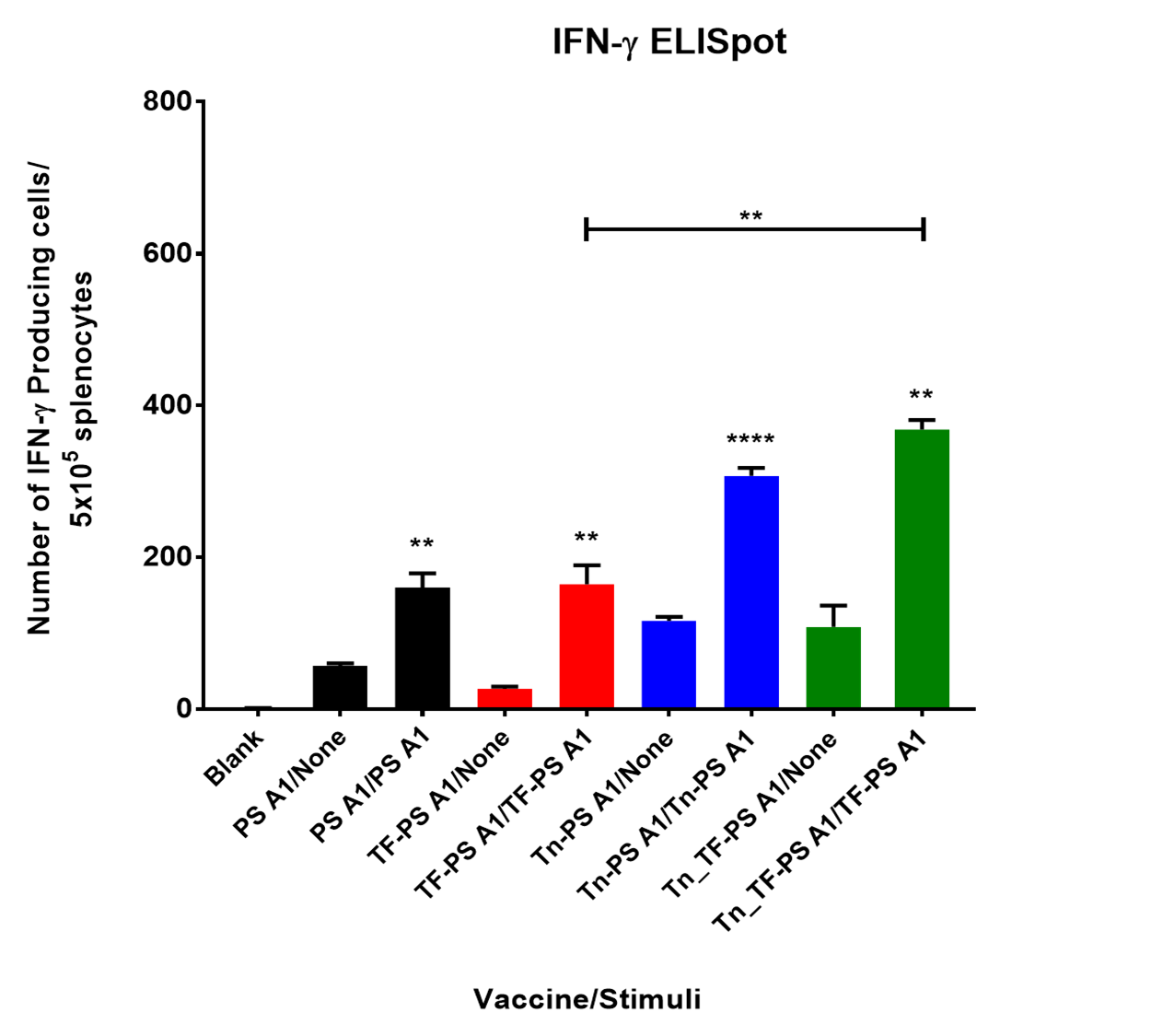
**

**Figure S6.** Representative pictures of IL-10 ELISpot followed by a bar chart.

**
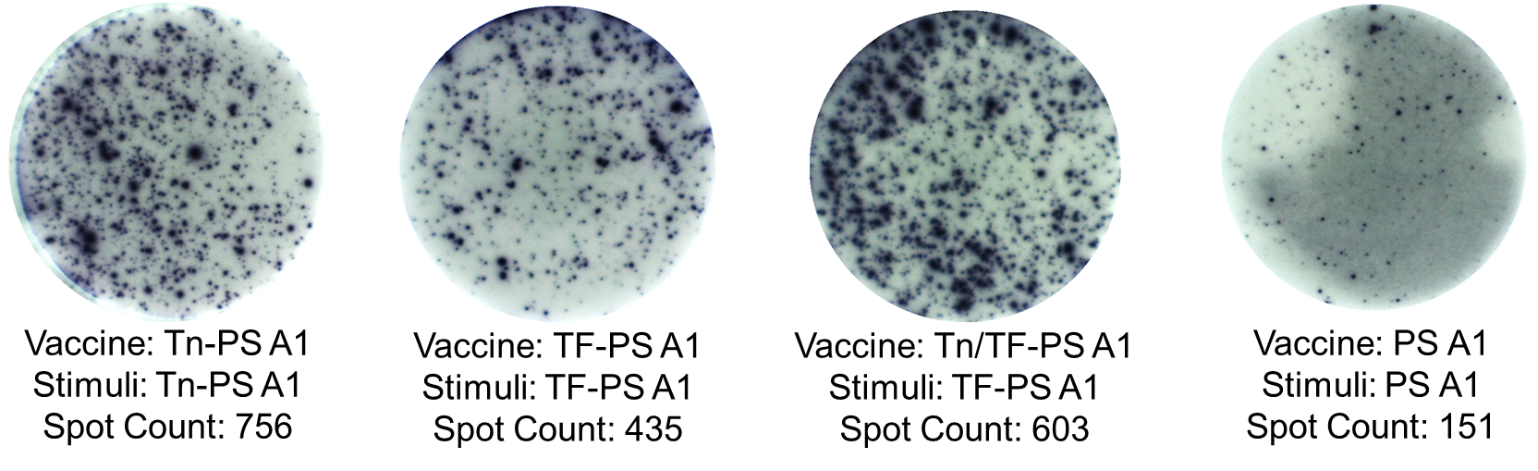
**

**
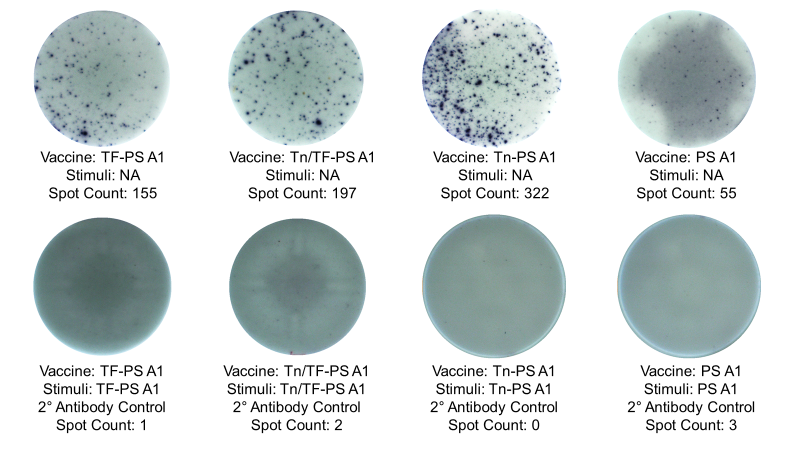
**

**
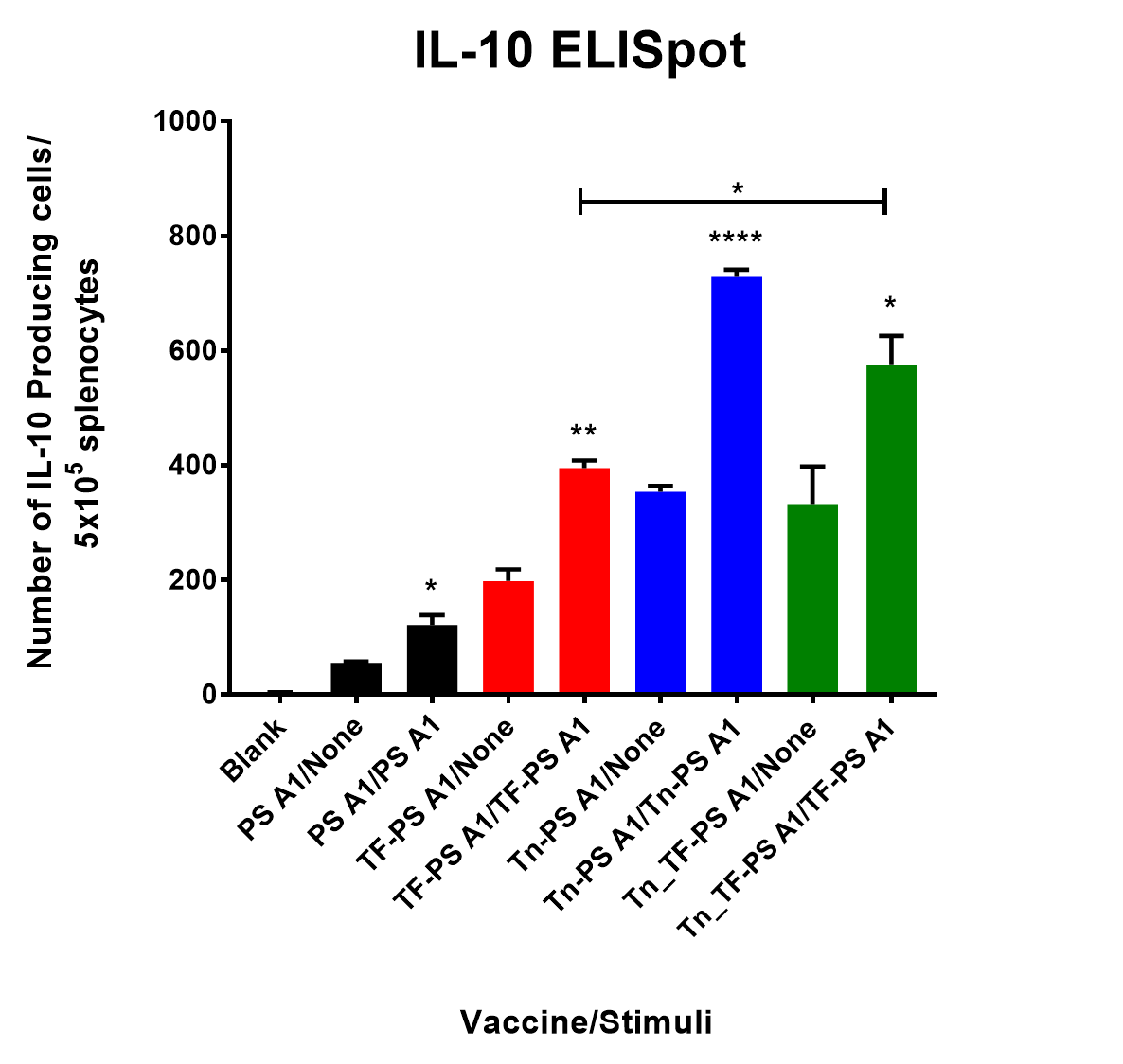
**
