# Supplementary material for: Enhanced Immune Response Against the Thomsen-Friedenreich Tumor Antigen Using a Bivalent Entirely Carbohydrate Conjugate
Source: Molecules. 2020 Mar 13;25(6):1319. doi: 10.3390/molecules25061319 (PMC7144725; doi:10.3390/molecules25061319)
Supplement: Supplementary file 1 [file molecules-25-01319-s001.zip › SI/Doc. S5-materials and methods.docx]

**Culture of Bacteroides fragilis and PS A1 isolation**

**Growth Conditions:** *Bacteroides fragilis* (ATCC 25285/NCTC 9343) was batch-cultured in 1 L Pyrex vessels containing PYG broth. 1 Liter of PYG broth was made by dissolving 20 g proteose peptone (Millipore Sigma 107229.1000), 5 g yeast extract (MP Biomedicals 3065-522) and 5 g NaCl in nanopure water. Once dissolved, the solution was autoclaved for sterility. After sterilization, the solution was sealed by septum and purged with nitrogen gas for 25 minutes to create an anaerobic environment. While purging with nitrogen, 5 mL of a sterile filtered solution containing 10% glucose, 10% dibasic potassium phosphate and 1% cysteine was injected into the broth (0.5 g glucose, 0.5 g dibasic potassium phosphate, 0.05 g cysteine). Additionally, 50 µL of a 0.5% vitamin K1 solution in absolute ethanol and 100 µL of a 0.5% hemin solution in 1M NaOH was injected during purging. After anaerobic conditions were established, a stock solution of *B. fragilis* in PYG broth and glycerol was inoculated into the vessel. The vessel was then set in an incubator at 37 ⁰C for 48 hours. After confluency of culture, an aliquot was collected for further expansion and the rest of the culture was centrifuged (6000 rpm, 20 minutes) to form a bacterial cell pellet. The broth was decanted into a bleach solution, while the pellet was suspended in a minimal amount PBS buffer. The cell suspension was stored at -80 ⁰C until 20 L of culture was processed.

**Isolation:** *B. fragilis*, suspended in 250 mL of PBS buffer, was mixed 1:1 with 75% aqueous phenol and heated to 70 ⁰C with vigorous stirring for 30 minutes to lyse cells. The biphasic mixture was cooled with ice and the hydrophobic and hydrophilic phases were separated using a separatory funnel. The aqueous phase was collected and phenol was further removed from the aqueous layer by centrifugation. The aqueous phase was then concentrated overnight *via* airflow. The concentrated aqueous phase was then washed with diethyl ether three times. After washing with diethyl ether, the aqueous phase was dialyzed for 48 hours against DI water in a 10 KDMWCO dialysis tubing (Thermo Fisher 88245). The aqueous solution was then completely lyophilized. The lyophilized material was then reconstituted in 10 mL of a 10 mM Tris buffer with 15 mM NaCl (pH = 7.5) and seeded with 10 mg of RNaseA (Roche 10109142001). It was then incubated at 37 ⁰C overnight with gentle shaking. The mixture was then dialyzed and lyophilized. The lyophilized material was then reconstituted in 10 mL of a 0.1 M acetate buffer with 0.15 M NaCl (pH = 5.0) and seeded with 10 mg of DNase1 (Alfa Aesar J62229). It was then incubated at 37 ⁰C overnight with gentle shaking. The mixture was then dialyzed and lyophilized. The lyophilized material was then reconstituted in 10 mL 0.1 M Tris buffer with 10 mM CaCl_2_ and seeded with Pronase (Roche 10165921001). It was then incubated at 37 ⁰C overnight with gentle shaking. The mixture was then dialyzed and lyophilized. At this point, the lyophilized material was examined by NMR to ensure no aromatic signals were present. If aromatic peaks were observed, the material was subjected to RNase, DNase, and Pronase consecutively.

**Purification:** The crude capsular polysaccharide was then passed through a size exclusion column (Sephacryl S-400 GE 17-0609-01) using sodium deoxycholate buffer containing 0.5% sodium deoxycholate, 50 mM glycine, and 10 mM EDTA with the pH being adjusted to and held constant at 9.8 using 5 M NaOH. Fractions were monitored at 260 nm and 280 nm for any detection of nucleic acid or protein contaminants. Fractions that did not have >0.1 absorbance in these regions were collected, dialyzed, and lyophilized. Fractions that had >0.1 absorbance were pooled, dialyzed, lyophilized, and passed through the S-400 column in deoxycholate buffer again. Pure capsular polysaccharide was then subjected to 5% acetic acid at 80 ⁰C for 30 minutes and then neutralized with 1 M NaOH. Immediately, the sample was then passed through an anion exchange column (DEAE Sephacel GE 17-0500-01) with 50 mM Tris buffer (pH = 7.3) with increasing NaCl concentrations every ~10 mL ranging from (0 mM NaCl, 0.1 M NaCl, 0.5 M NaCl). Every 5 fractions (1 mL/fraction) were pooled, dialyzed, and lyophilized. Lyophilized materials were analyzed by NMR at 60 ⁰C to assess purity (Doc. S1).

**Tn-ONH_2_ (2)**

The preparation of Tn-ONH_2_ has been previously described [1]. Spectra are matched with previously reported data.

^1^H NMR (D_2_O, 600 MHz): δ_H_ 4.93 (1H, d, *J*=4.1 Hz, H1), 4.19 (1H, dd, *J*=4.1, 11.3 Hz, H2), 3.96 (2H, m, H4 and H5), 3.82 (1H, dd, *J*=3.2, 11.3 Hz, H3), 3.75 (2H, m, H6 and H6’) 2.02 (3H, s, NHAc). ^13^C NMR (D_2_O ,150 MHz): δ_C_ 174.6, 100.6, 70.1, 68.3, 67.5, 61.1, 49.1, 21.8. LRMS:ESI [M+(Na)^+^] calcd for 259.1 found 259.1.

**TF-ONH_2_ (3)**

The preparation of TF-ONH_2_ has been previously described [2]. Spectra are matched with previously reported data.

^1^H NMR (D_2_O, 600 MHz): δ_H_ 4.92 (1H, d, *J*=4.2 Hz, GalNAc H1), 4.42 (1H, d, *J*=7.8 Hz, Gal H1), 4.36 (1H, dd, *J*=4.0, 11.2, GalNAc H2), 4.21 (1H, d, *J*=2.7, GalNAc H4), 4.00 (1H, dd, *J*=4.5, 7.2, GalNAc H5), 3.95 (1H, dd, *J*=3.0, 11.2, GalNAc H3), 3.87 (1H, d, *J*=3.5, Gal H4), 3.74 (4H, m, GalNAc H6, H6’, Gal H6, H6’), 3.60 (2H, m, Gal H3, H5), 3.48 (1H, dd, *J*=7.5, 9.7, Gal H2), 2.00 (3H, s, NHAc). ^13^C NMR (D_2_O ,150 MHz): δ_C_ 174.6, 104,6, 100.7, 76.8, 74.9, 72.4, 70.6, 70.5, 68.6, 68.5, 61.0, 60.9, 47.8, 21.9. LRMS:ESI [M+(Na)^+^] calcd for 421.1 found 421.2.

**Synthesis of Tn-PS A1 (4a)**

The preparation of Tn-PS A1 has been previously described [1]. Sec Doc. S5 for characterization of Tn-PS A1.

**Synthesis of TF-PS A1 (4b)**

The preparation of TF-PS A1 has been previously described [2]. See Doc S5 for characterization of TF-PS A1.

**Synthesis of Tn-TF-PS A1 (4c)**

55 μL of a 10 mM solution of NaIO_4_ was used to oxidize 1 mg of PS A1 in 0.5 mL of 0.1 M NaOAc buffer (pH 5.2) for 90 min in the dark. An excess of KCl was used to quench NaIO_4_. A 1:1 molar ratio of Tn-ONH_2_ (**2**) to TF-ONH_2_ (**3**) (1.1 mg and 1.7 mg respectively) were allowed to react with oxidized PS A1 for 24 hours followed by overnight dialysis using 10 kDa MWCO SnakeSkin™ tubing. See Doc. S5 for characterization off Tn-TF-PS A1.

**Biotinylated PS A1 (5a-d)**

1.0 mg of PS A1 (**1**), Tn-PS A1 (**4a**), TF-PS A1 (**4b**), or Tn-TF-PS A1 (**4c**) was reacted with 0.5 mg of sulfo-NHS-biotin (100X equivalents; b2100, ProteoChem) in 0.5 mL of 1X PBS buffer (pH 7.4) for 24 h at room temperature. The PS A1 probes were dialyzed, lyophilized, and reconstituted in 1X PBS buffer (with CaCl_2_/MgCl_2_, pH 7.2) at a concentration of 1 mg mL^-1^.

***S*-(3-oxopropyl) ethanethioate (mercaptoaldehyde) (8)**

A catalytic amount of piperidine (5.0 µL) was added to 0.5 mL acrolein (**6**) at 0 ^o^C. Then, 0.52 mL of thioacetic acid (**7**) was added dropwise over a period of 30 minutes. The reaction was carried out for 12 hours and the reaction mixture was then concentrated under vacuum and purified by column chromatography using 30% EtOAc/70% DCM as the eluent to give mercaptoaldehyde (**8**) in 95% yield.

^1^H NMR (CDCl_3_, 600MHz): δ_H_ 9.67 (1H, d, *J*=1.0 Hz), 3.03 (2H, t, *J*=1.0 Hz), 2.73 (2H, t, *J*=1.0 Hz), 2.25 (3H, d, *J*=1.0 Hz); ^13^C NMR (CDCl_3_,150 MHz): δ_C_ 200.1, 195.6, 43.8, 30.7, 21.7. LRMS:ESI [M+(Na)^+^] calcd for 155.01 found 155.0.

**Intermediate 9**

Aminooxy Tn (**2**) (5.0 mg) was reacted with 2.8 mg of mercaptoaldehyde (**8**) for 18 h in sodium acetate buffer (pH 5.5) at room temperature. The Tn-linker (**9**) was then purified using a Sephadex G-10 biogel column and deionized/distilled H_2_O as the eluent. TLC was used to confirm those fractions containing the Tn-linker (**9**). The fractions containing the Tn-linker were collected in 50 mL centrifuge tubes, frozen then lyophilized.

^1^H NMR (D_2_O ,600MHz): δ_H_ 7.51 - 7.54 (1H, m), 6.89 - 6.92 (1H, m), 5.37 (1H, d, *J*=3.7 Hz), 5.28 - 5.30 (1H, m), 4.15 - 4.21 (1H, m), 3.90 - 3.95 (1H, m), 3.80 - 3.88 (2H, m), 3.59 - 3.68 (2H, m), 2.92 - 3.03 (2H, m), 2.58 - 2.71 (1H, m), 2.43 (1H, dt, *J*=9.5, 6.2, 2.9 Hz), 2.24 - 2.29 (2H, m), 1.91 - 1.97 (3H, m), 1.80 (1H, br). ^13^C NMR (D_2_O ,150 MHz): δ_C_ 200.9, 174.5, 154.6, 104.7, 98.7, 76.8, 74.9, 72.4, 70.5, 68.5, 60.8, 47.6, 30.0, 29.3, 25.8, 25.4, 25.0, 23.2, 21.9. LRMS:ESI [M+(Na)^+^] calcd for 373.10 found 373.1.

**Intermediate 10**

Aminooxy TF (**3**) (5.0 mg) was reacted with 1.7 mg of mercaptoaldehyde (**8**) for 18 h in sodium acetate buffer (pH 5.5) at room temperature. The TF-linker was then purified using a Sephadex G-10 biogel column and deionized/distilled H_2_O as the eluent. TLC was used to confirm those fractions containing the TF-linker (**10**). The fractions containing the TF-linker were collected in 50 mL centrifuge tubes, frozen then lyophilized.

^1^H NMR (D_2_O, 600MHz): δ_H_ 7.53 (1H, t, *J*=6.2 Hz), 6.91 (1H, s), 5.37 (1H, d, *J*=4.0 Hz), 5.28 (1H, d, *J*=4.0 Hz), 4.40 (1H, d, *J*=8.1 Hz), 4.32 - 4.39 (1H, m), 4.19 (1H, d, *J*=2.9 Hz), 4.16 (1H, d, *J*=2.9 Hz), 3.95 (1H, dt, *J*=11.2, 2.7 Hz), 3.84 - 3.89 (1H, m), 3.80 (1H, d, *J*=2.9 Hz), 3.59 - 3.69 (2H, m), 3.49 - 3.58 (1H, m), 3.42 (1H, dt, *J*=9.9, 7.7 Hz), 2.92 - 3.05 (1H, m), 2.57 - 2.75 (1H, m), 2.44 (1H, q, *J*=6.6 Hz), 2.33 (1H, s), 2.24 - 2.28 (1H, m), 1.91 (1H, s), 1.80 (1H, s). ^13^C NMR (D_2_O ,150 MHz): δ_C_ 200.9, 174.5, 154.6, 104.7, 98.7, 76.8, 74.9, 72.4, 70.5, 68.5, 60.8, 47.6, 30.0, 29.3, 25.8, 25.4, 25.0, 23.2, 21.9. LRMS:ESI [M+(Na)^+^] calcd for 535.15 found 535.1.

**Maleimide functionilzed BSA (11)**

BSA (1.0 mg) was dissolved in 300 µL of 1X PBS buffer with 1 mM EDTA (pH 7.2). This solution was then reacted with 100 µL of a 2 mM 3-(maleimido)propionic acid *N*-hydroxysuccinimide solution in DMF. Excess 3-(maleimido)propionate was removed by centrifugal ultrafiltration (Vivaspin® 6 MWCO 10 kDa) and washed three times with 5 mL of 1X PBS buffer containing 1 mM EDTA (pH 7.2). Conjugation was analyzed by MALDI-TOF and determined to be M/Z = 71686.967. Mass loading was calculated using the following equation: (MW of BSA-maleimide – MW of BSA (M/Z = 66430))/(MW of maleimide linker). Based on the molecular weight, we were able to determine that there were 34 molecules of maleimide linked to BSA (7.3% by mass).

**Tn-BSA (12)**

Tn-linker (**9**) (2.5 mg) was deacetylated under Zemplen’s conditions of NaOMe in methanol. The reaction was quenched with DOWEX 50W x 8-100 ion exchange resin after 30 minutes of reaction time. The solution was then filtered and concentrated under reduced pressure. The deacetylated Tn-linker was dissolved in 0.1 mL of 1X PBS buffer with 1 mM EDTA (pH 7.2). It was then added to a solution of 1.0 mg BSA-maleimide (**11**) that was dissolved in 0.2 mL 1X PBS buffer. Conjugation was analyzed using MALDI-TOF (M/Z = 80119.571). Mass loading was calculated using the following equation: (MW of Tn-BSA – MW of BSA-maleimide)/(MW of Tn-linker). From this method, we determined that there were 27 molecules of Tn-linker conjugated per BSA-maleimide (10.5% by mass).

**TF-BSA (13)**

TF-linker (**10**) (2.5 mg) was deacetylated under Zemplen’s conditions of NaOMe in methanol. The reaction was quenched with DOWEX 50W x 8-100 ion exchange resin after 30 min of reaction time. The solution was then filtered and concentrated under reduced pressure. The deacetylated TF-linker was dissolved in 0.1 mL of 1X PBS buffer with 1 mM EDTA (pH 7.2). It was then added to a solution of 1.0 mg BSA-maleimide (**11**) that was dissolved in 0.2 mL 1X PBS buffer. Conjugation was analyzed by MALDI-TOF (M/Z = 78273.845). Mass loading was calculated using the following equation: (MW of TF-BSA – MW of BSA-maleimide)/(MW of TF-linker). We determined there were 14 molecules of TF-linker conjugated per BSA-maleimide (8.4% by mass).

**NMR Analysis**

NMR analyses were performed using a Bruker Avance III 600 MHz spectrometer. All PS A1 and PS A1 conjugate spectra were collected at a probe temperature of 60 °C. Monomer antigen spectra were collected at 22 °C. ^1^H spectra were obtained using the *zg30* pulse program (D1 = 1 sec., AQ = 2.7 sec). ^13^C spectra were obtained using the *zpg30* pulse program (D1 = 2 sec., AQ = 0.9 sec). COSY spectra were obtained using the *cosygpppqf* pulse program (D1 = 2 sec, AQ = 0.38 sec (for Tn-ONH2 and TF-ONH2) 0.22 sec (PS A1 conjugates). 1D TOCSY spectra were obtained using the *selmlgp* pulse program (D1 = 2 sec, AQ = 2.7 sec, D9 = 0.120 sec).

**Cytokine Profile**

For the primary method of *in vitro* splenic cytokine release assay, spleens were aseptically removed from immunized mice, then cultured in 10% FBS (v/v) RPMI 1640, and stimulated for 48 h in individual 96 well-plates in the presence of PS A1, Tn-PS A1, TF-PS A1, and Tn-TF-PS A1 constructs. The experiments were performed in triplicate. Groups of mice (n=3) were injected on days 0, 21, 35 and spleens were collected 10 days after the final injection. The spleens were homogenized into single cell suspensions and then strained over a 40 μm cell strainer into a 50 mL tube. The cells were centrifuged at 1000 rpm for 10 minutes. The cell pellet was treated with red blood cell lysis buffer (3 mL) for 3-4 min on ice. Then diluted with 35 mL of cell culture media and centrifuged at 1000 rpm for 10 min to pellet the cells and remove the buffer. The cells were strained over a 40 μm strainer to remove dead clumps of cells. The spleen cells were counted and diluted to 1.0 x 10^6^ cells mL^-1^ and then seeded onto 96 well-plates. For stimulation of the splenocytes, 20 μg of TACA-PS A1 conjugates, 20 μg of PS A1, or PBS buffer (negative control) were added to individual sections of the well-plates. In a separate 96-well plate, rabbit anti-mouse cytokine capture antibodies (IL-10, IL-17, INF-γ; Peprotech) were diluted to a concentration of 1.0 μg mL^-1^ (PBS buffer; pH = 7.2). 100 μL were added to the appropriate wells and incubated overnight at room temperature to allow antibody to bind to the plate. The plates were washed four times with 300 μL washing buffer (PBS with 0.05% Tween 20 (v/v)) and blocked with 300 μL blocking buffer (PBS with 1% BSA (w/v)) for 1 hour at room temperature, followed by another washing step. Then 100 μL of cell culture supernatants or cytokine standards (serially diluted from 0.01 μg mL^-1^ – 0.00 μg mL^-1^ in PBS with 0.05% Tween-20 (v/v), 0.1% BSA (w/v)) were incubated on the antibody plate for 2 hours at room temperature and washed. 100 μL of biotin conjugated detection antibodies, specific for respective cytokines, at 0.5 μg mL^-1^ (PBS with 0.05% Tween-20 (v/v), 0.1% BSA (w/v)) were added to each well and incubated at room temperature for 2 h followed by washing. Streptavidin-HRP conjugate was diluted 1:20,000 (PBS with 0.05% Tween-20 (v/v), 0.1% BSA (w/v)) and 100 μL were added per well and incubated for 30 min followed by washing. Colorimetric detection of streptavidin-HRP conjugates was conducted using 100 μL of TMB substrate solution for 20 min and optical densities were read at 450 nm with a wavelength correction at 620 nm.

**ELISpot**

Three C57BL/6 mice were immunized with respective TACA-PS A1 plus SAS and splenocytes were isolated following our described procedures above. Total cell counts were determined and then they were plated in corresponding wells at a cell concentration of 5.0 x 10^5^ cells well^-1^. All cells were stimulated with 20 μg of stimuli (sterile filtered DPBS for negative controls) and incubated at 37 °C and 5% CO_2_ for 36 h. For a secondary antibody control, the secondary antibody solution was replaced with sterile filtered DPBS. The ELISpot procedure was performed per the Kit Protocol (MABTECH; 3321-4APW-2 (IFN-γ), 3521-4APW-2 (IL-17A), 3432-4APW-2 (IL-10)). Spot counts were obtained using MVS Pacific QuantiHub Color 4.1 ELISpot plate reader. Spots were counted using the automated spot counting algorithm available in the QuantiHub 4.1 software. Confluent wells above the upper limit of quantification were assumed to contain at least 600 spots/well (IFN-γ) and 800 spots/well (IL-10) [3]. These wells were not used in any statistical analysis.

**References**

1. De Silva, R. A.; Wang, Q.; Chidley, T.; Appulage, D. K.; Andreana, P. R. Immunological Response from an Entirely Carbohydrate Antigen: Design of Synthetic Vaccines Based on Tn−PS A1 Conjugates *J. Am. Chem. Soc.* **2009**, *131,* 9622-9623.
2. Bourgault, J. P.; Trabbic, K. R.; Shi, M.; Andreana, P. R. Synthesis of the tumor associative alpha-aminooxy disaccharide of the TF antigen and its conjugation to a polysaccharide immune stimulant *Org. Biomol. Chem.* **2014**, *12,* 1699-1702.
3. Sundararaman, S.; Karulin, A. Y.; Ansari, T.; BenHamouda, N.; Gottwein, J.; Laxmanan, S.; Levine, S. M.; Loffredo, J. T.; McArdle, S.; Neudoerfl, C.; Roen, D.; Silina, K.; Welch, M.; Lehmann, P. V. High Reproducibility of ELISPOT Counts from Nine Different Laboratories *Cells* **2015**, *4,* 21-39.
